# Sustainable human population density in Western Europe between 560.000 and 360.000 years ago

**DOI:** 10.1038/s41598-022-10642-w

**Published:** 2022-04-28

**Authors:** Jesús Rodríguez, Christian Willmes, Christian Sommer, Ana Mateos

**Affiliations:** 1 Centro Nacional de Investigación sobre la Evolución Humana (CENIEH), Paseo Sierra de Atapuerca 3, 09002 Burgos, Spain; 2 Institute of Geography, University of Cologne, 50923 Cologne, Germany; 3 The Role of Culture in Early Expansions of Humans, Research Area Geography, Heidelberg Academy of Sciences and Humanities at the University of Tübingen, Rümelinstr. 19-23, 72070 Tübingen, Germany

**Keywords:** Archaeology, Palaeontology, Ecological modelling, Palaeoecology

## Abstract

The time period between 560 and 360 ka (MIS14 to MIS11) was critical for the evolution of the Neanderthal lineage and the appearance of Levallois technology in Europe. The shifts in the distribution of the human populations, driven by cyclical climate changes, are generally accepted to have played major roles in both processes. We used a dataset of palaeoclimate maps and a species distribution model to reconstruct the changes in the area of Western Europe with suitable environmental conditions for humans during 11 time intervals of the MIS14 to MIS 11 period. Eventually, the maximum sustainable human population within the suitable area during each time interval was estimated by extrapolating the relationship observed between recent hunter-gatherer population density and net primary productivity and applying it to the past. Contrary to common assumptions, our results showed the three Mediterranean Peninsulas were not the only region suitable for humans during the glacial periods. The estimated total sustainable population of Western Europe from MIS14 to MIS11 oscillated between 13,000 and 25,000 individuals. These results offer a new theoretical scenario to develop models and hypotheses to explain cultural and biological evolution during the Middle Pleistocene in Western Europe.

## Introduction

The Middle Pleistocene was a critical period for human evolution in Europe, marked by cyclic climate instability. Since 640 ka onwards shifts in the climate occurring every 100 ka caused a succession of glaciations and interglacials that are usually referred to the Marine Isotope Stages (MIS) and substages^1^. The four stages correlated with the 560 ka to 360 ka interval are MIS14 to MIS11. The MIS12 (Anglian or Elsterian glaciation) is considered a major turning point for the human occupation of Europe, marked by an increased abundance of archaeological data across Eurasia after this period^2^. Cultural complexity increased in Western Europe from MIS12 onwards, with the onset of Levallois, the spread of bifacial technology, and more complex resource management^2,3^. Moreover, the palaeontological evidence and many palaeogenetic analyses suggest that the earliest Neanderthal features appeared across Western Europe around 600–450 ka (roughly MIS15–MIS12), and the evolutionary process that gave rise to Neanderthals seems to have been complex^4–9^. Most models dealing with cultural and biological changes in Middle Pleistocene Europe include the effect of repeated shifts in the latitudinal distribution of humans, promoted by the cyclic climate oscillations characteristic of this period. It is generally assumed in these models that the distribution range of the European hominins expanded northwards during the interglacial stages and collapsed towards the Iberian, Apennine (or Italian), and Balkan Peninsulas during the glacial stages^1,8^. However, a precise delimitation of the range occupied by humans during each glacial and interglacial stage is currently impossible because of the relative scarcity of sites from this period. Arguably, the range expansions and contractions affected not only the spatial distribution of hominins but also the sizes of their census and effective populations. It is debatable whether increments in population size affected cultural changes in the Palaeolithic^10–12^, but oscillations in population size likely influenced biological evolution by modifying the frequency of some alleles and favouring the spread of certain genotypes by random genetic drift^13^.

Several different approaches have been developed to estimate the population density of Palaeolithic groups. These approaches include estimates derived from comparing the relative frequencies of different taxa between fossil assemblages and recent ecosystems^14^, extrapolation of ethnographic data^15^ combined with the observed frequency of archaeological sites^16–18^ or with the estimated size of the inhabitable area^19^, mathematical models simulating resource availability and competition in past environments^20^, and climate envelope models^21^. In addition to estimates of ancestral population size based on genetic data^22^, models producing continuous predictions of population density over a continent have proven a feasible approach for estimating past population sizes^21^. However, estimations of population size specific to the MIS14–MIS11 period in Europe are not available.

This study aimed to make two key contributions to the debate on the dynamics of cultural and biological evolution in Middle Pleistocene Europe. First, we present estimations of the potential human distribution in Western Europe during 11 cold and warm intervals of the MIS14–MIS11 period^23^ based on the lettering scheme for marine isotope substages^24^. Second, we provide human population size estimations that would have been sustained by Western European ecosystems during these 11 intervals. Our focus was on Western Europe because it has the most complete palaeontological and archaeological records for the Middle Pleistocene and, consequently, most models of cultural and biological evolution were developed specifically for this area^2,8,15,25^.

**Figure 1:**
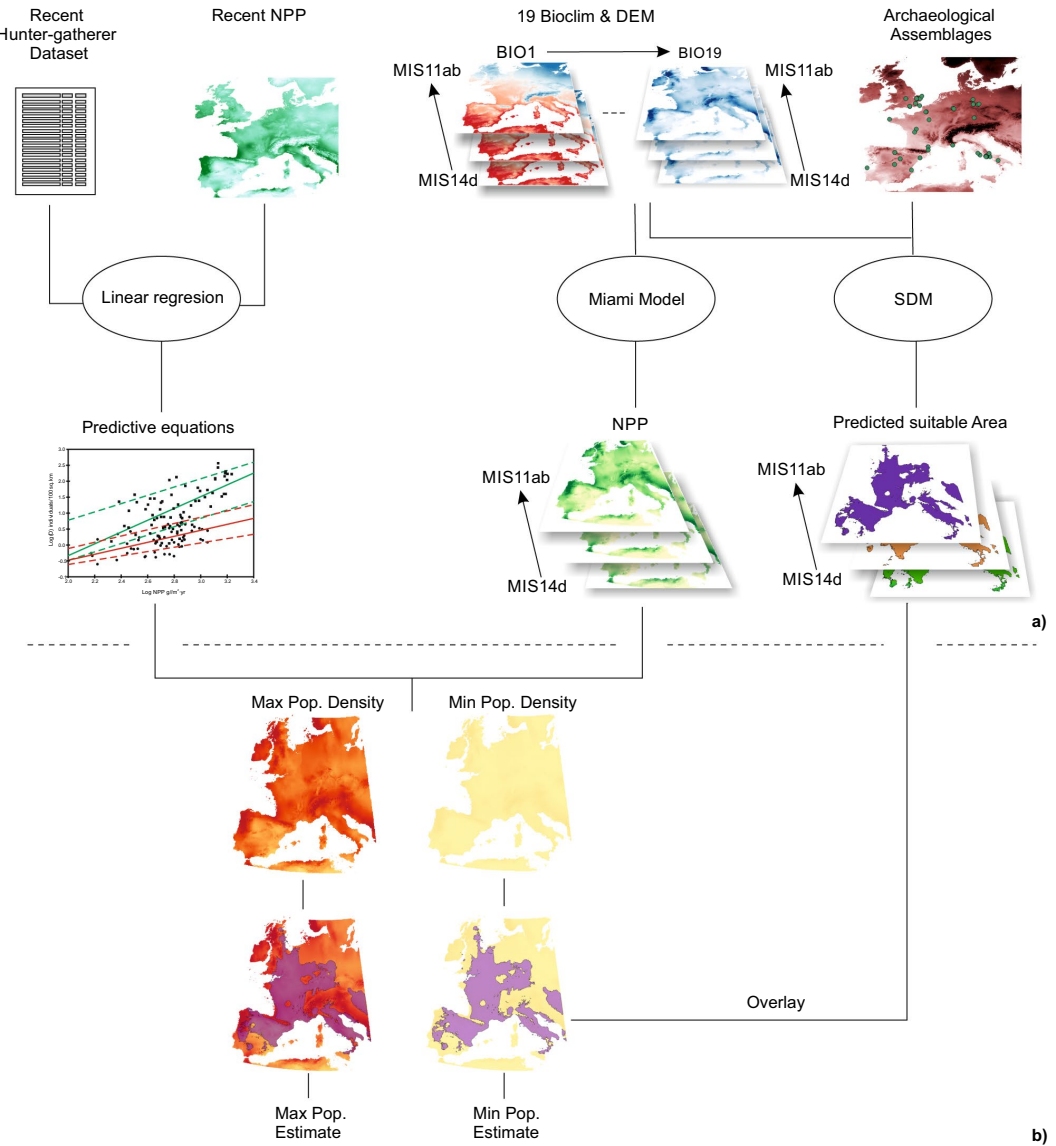
Overview of the methodological approach for estimating total sustainable human populations in Western Europe from MIS 14 to MIS11. (**a**) Population density of recent hunter-gatherer groups and net primary productivity (NPP), both obtained from published sources (see “Methods”), were combined to obtain predictive equations of population density from NPP. 19 bioclimatic variables obtained from the Oscillayers dataset (see “Methods”) and elevation obtained from a digital elevation model were used as predictors in a species distribution model (SDM) to obtain a map of the suitable area for humans during each interval (see “Methods”). Presence data for the SDM were obtained from a dataset of archaeological sites dated to the period 560 ka to 360 ka, NPP maps for the interval were obtained from mean annual temperature (BIO1) and annual precipitation (BIO12) by applying the Miami model (see “Methods”). (**b**) The following procedure was repeated for each interval. Maximum and minimum population density maps were obtained by applying the predictive equations to the NPP map. Eventually, the total sustainable population in the interval was estimated by multiplying the average population density inside the predicted distribution range of humans by the size of the area (see “Methods”). The maps were created in QGIS 3.22 (https://www.qgis.org).

We applied an ecological niche modelling approach to estimate the potential distribution of humans for each interval based on a dataset of 68 archaeological assemblages from Western Europe, confidentially dated to this period (see “Methods” and Fig. 1). Palaeoclimate data for each interval of the MIS14–MIS11 period were obtained from the Oscillayers palaeoclimatic database^26,27^. Following other studies that modelled human distributions from archaeological data^28,29^, we assumed the Eltonian hypothesis^30^, which considers that the distribution of a species, hominins in our case, is determined by abiotic factors and minimally affected by biotic interactions. Thus, we selected the six most informative predictors from 19 bioclimatic variables and elevation (see “Methods”).

Net primary productivity (NPP) is a major determinant of hunter-gatherer population density, especially in the Holarctic^31^. Thus, we used a dataset of recent hunter-gatherer populations^32^ from Holarctic zones and NPP data^31^ to obtain a predictive equation for the maximum sustainable hunter-gatherer population density. It should be noted that, although our approach is a simple projection of the relationship observed between ethnographic hunter-gatherer population density and NPP, there are strong theoretical reasons to assume that there is a causal relationship between environmental productivity and hunter-gatherer population density^31,33^. The population density at which a species may occur in a particular environment is limited by the availability of trophic resources, which is directly dependent on NPP^34^. Since it is a well-known ecological rule that secondary consumers in natural communities have lower population densities than primary consumers^35,36^, a different relationship between population density and NPP in hunter-gatherer groups relying on different procurement strategies may be expected. Recent hunter-gatherer groups may be classified as ‘hunters’, ‘gatherers’ or ‘fishers’ according to their main procurement strategy^32^, although most of them use all three types of resources to some degree. Although the oldest evidence of fish consumption dates back to 1.95 Ma.^37^, foragers would have required more advanced technology than was available during the Lower Palaeolithic^38^ to rely on fishing as the main procurement strategy. Indeed, all archaeological assemblages dated to the Lower Palaeolithic that have faunal remains have provided overwhelming evidence for mammal consumption. In contrast, the evidence of aquatic resource consumption is very scarce for sites older than 160 ka^39^. Furthermore, well-preserved wooden hunting spears dated to around 300 ka were found at Schöningen^40^, but there is no direct or indirect evidence of nets or harpoons until the Upper Palaeolithic. Moreover, aquatic resources seem to have substituted for meat for contemporary hunter-gatherers who mainly rely on aquatic resources^41^. Thus, we excluded ‘fishers’ from our model to estimate population density in the Lower Palaeolithic and explored the relationships between NPP and density for ‘hunters’ and ‘gatherers’ separately (see “Methods”).

We estimated the sustainable population of Western Europe during each interval by multiplying the average hunter-gatherer sustainable population density in the area with suitable environmental conditions for humans by the size of the area (Fig. 1b).

## Results

### Relationship between NPP and population density

**Figure 2:**
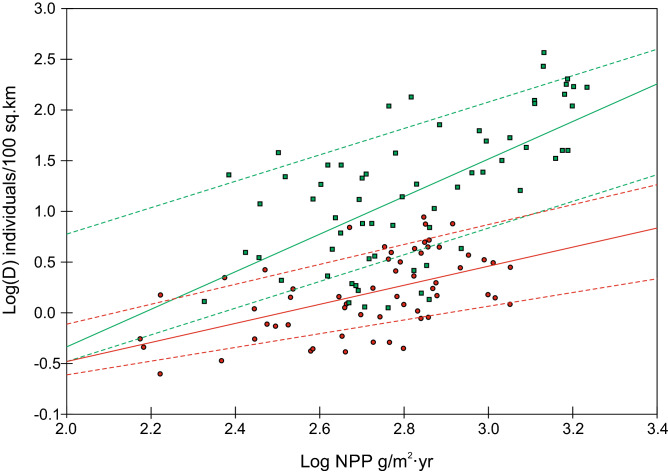
Relationship between net primary production (NPP) and hunter-gatherer population density (D). Green squares represent groups classified as ‘gatherers’ and red circles correspond to ‘hunters’. The solid lines are the regression equations fitted to the total sample of ‘gatherers’ (green) and the total sample of ‘hunters’ (red). Dashed lines are the regression equations to the upper and lower limit of the distribution of ‘gatherers’ (green) and ‘hunters’ (red), respectively.

As already noted by other authors^31^, there is a significant relationship between NPP and population density for the entire sample of hunter-gatherer groups (Spearman’s rho = 0.68; p < 0.05, N = 215). Interestingly, the relationship is markedly different based on the main subsistence strategy (Table 1, Fig. 2). Excluding horseback hunters from the dataset did not significantly change the slope of the relationship (F = 1.099, p = 0.7291; t = − 1.061 p = 0.2911, N = 101, see Supplementary Figure S1).

**Table 1:** Simple linear regression of log-transformed hunter-gatherer population density (D) on log-transformed net primary productivity (LOGNPP). Regression equations were fitted to the whole sample (average) and the upper and lower limit of the distribution for each procurement strategy.

		Equation	r	p	r^2^	N
Hunters	Average	LogD = − 2.362 + 0.9427*LOGNPP	0.5336	0.00001	0.28	60
	Lower limit	LogD = − 1.9562 + 0.6751*LOGNPP	0.6800	0.0150	0.46	12
	Upper limit	LogD = − 2.0659 + 0.9805*LOGNPP	0.8056	0.0049	0.65	10
Gatherers	Average	LogD = − 4.0253 + 1.8485*LOGNPP	0.6341	0.00000	0.40	65
	Lower limit	LogD = − 3.0816 + 1.3073*LOGNPP	0.7048	0.0340	0.50	9
	Upper limit	LogD = − 1.819 + 1.3*LOGNPP	0.7823	0.0127	0.61	9

The slopes of the lines show that ‘gatherers’ were able to sustain higher population densities than ‘hunters’ at a given amount of NPP. This aligns with the observation that ‘hunters’ require larger home ranges than “gatherers” ^33^.

### Ecological Niche model

**Figure 3:**
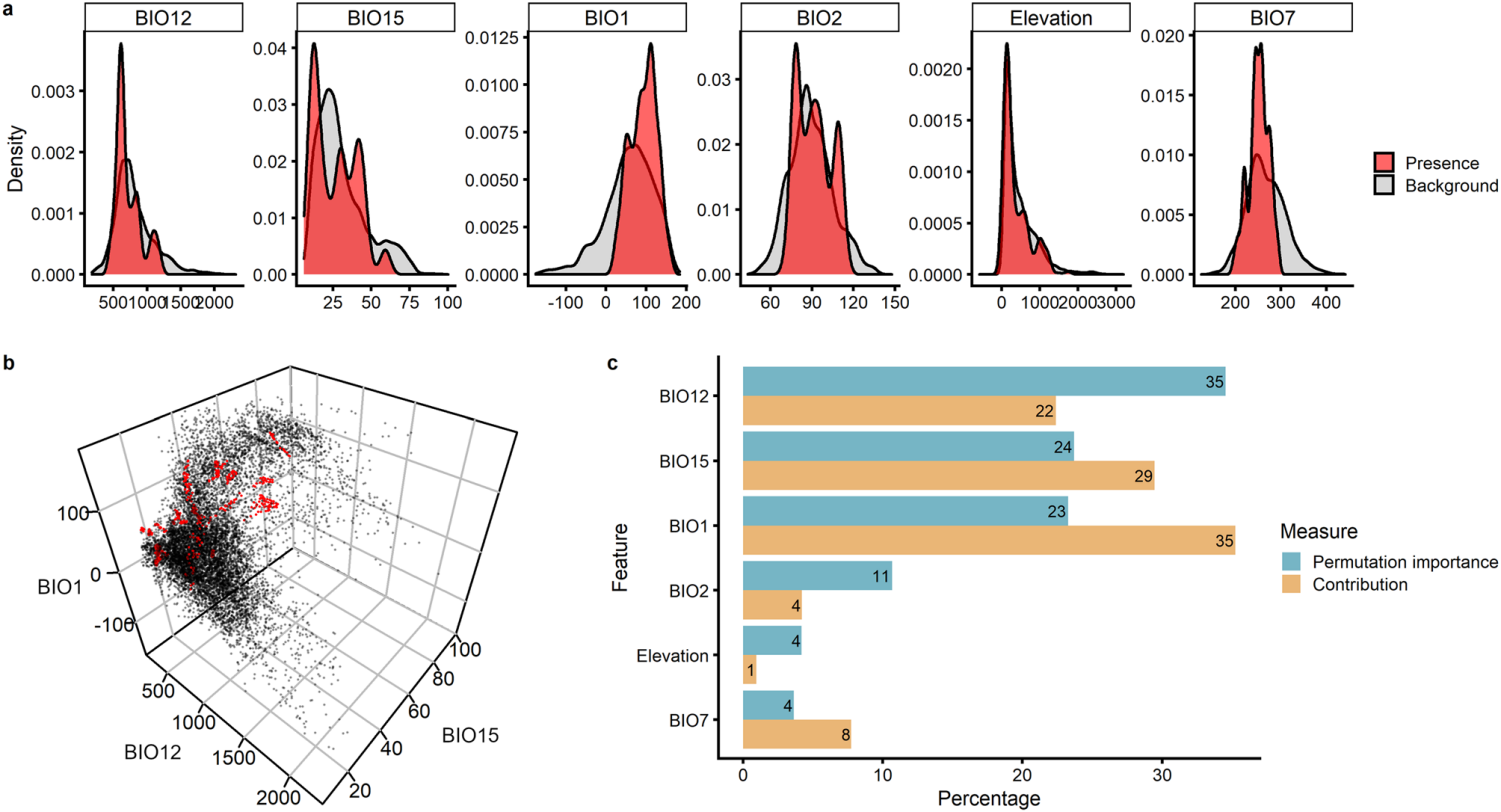
The human ecological niche throughout MIS 11–14 according to the selected SDM. (**a**) Comparison of densities at confirmed presence points (n = 644; red) with a random sample (n = 10,000; grey) of the overall environment. (**b**) Depictions of the human niche (red) within the environment (grey) in a covariate space with the 3 most important covariates. (**c**) Ranking of covariate importance based on the metrics permutation importance (blue) and model contribution (yellow).

Comparing different model candidates highlighted the necessity of calibrating the species distribution model (SDM) for individual species^42–44^. Our results (Fig. 3) showed that more complex feature classes led to considerably better performance in validation metrics, such as the increment in the Akaike information criterion for small sample sizes (∆AICc) and the area under the receiver-operator curve (AUC). However, we interpreted this as an effect of overfitting, because the complex models showed much higher omission rates in the testing subsample. Therefore, we prefer a compromise model that delivers reasonable values for all indicators considered. Furthermore, we could not find ‘peak performance’ at the default regression multiplier (RM) of 1.0, but rather observed a trend indicating that lower RMs produced better results than higher RMs. This observation contradicts other studies^43–45^ that recommend higher RMs, which impose higher penalties to include covariates, and thus, simplify the model. However, such studies used larger and unreduced covariate sets that did not undergo a preselection process (see “Methods”), so collinear and correlated covariates were likely penalised through the higher RM. We interpreted this as an advantage of our statistical preprocessing, which retained only covariates with high individual information content, and thus, were able to inform more complex models with lower penalties.

The most informative variables for niche estimation were annual precipitation (BIO12), precipitation seasonality (BIO15), and mean annual temperature (BIO1), while mean diurnal temperature range (BIO2), elevation, and temperature annual range (BIO7) played minor roles (Fig. 3c). Thereafter, the major limiting factors for the human niche were extremely dry (BIO12 < 300 mm) and wet (BIO12 > 1200 mm) conditions, strong precipitation seasonality (BIO15 > 65), and mean annual temperatures below 0 °C (Fig. 3a and b).

**Figure 4:**
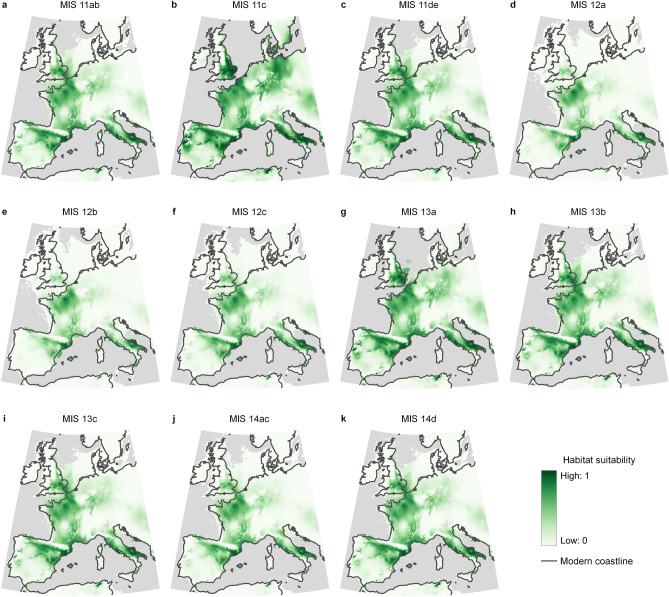
Variation in the area suitable for humans during the MIS14 to MIS11 period. The maps were created in QGIS 3.22 (https://www.qgis.org).

The niche model showed great stability in the extent and distribution of the permanent core areas suitable for hominins from MIS14 to MIS11 and a temporally varying periphery (Supplementary Figure S2). The most variable areas corresponded to the North Sea, the Jutland Peninsula, and adjacent territories. Moreover, the region east of the Oder River was only suitable for humans during MIS11c (Fig. 4). This variability was determined by the unsuitability of the northern region during the glacial stages, especially during MIS12. The niche model predicts a continuous suitable area connecting the southern peninsulas to Britain through France and West Germany for most of the time. Britain’s connection with the continent was only interrupted during the MIS11c and MIS13a. The areas predicted to be non-suitable throughout the entire MIS14 to MIS11 period were the high-altitude regions of the Pyrenees and the Alps; Brittany; two small spots in the Ardennes and central France; and a large part of the Iberian Peninsula, including the southern half and the northwest corner. The exclusion of a large area of the Iberian Peninsula might be explained by the unique climatic conditions of this region and the absence of sites confidentially dated to this period.

### Total population from MIS14 to MIS11 in Western Europe

**Figure 5:**
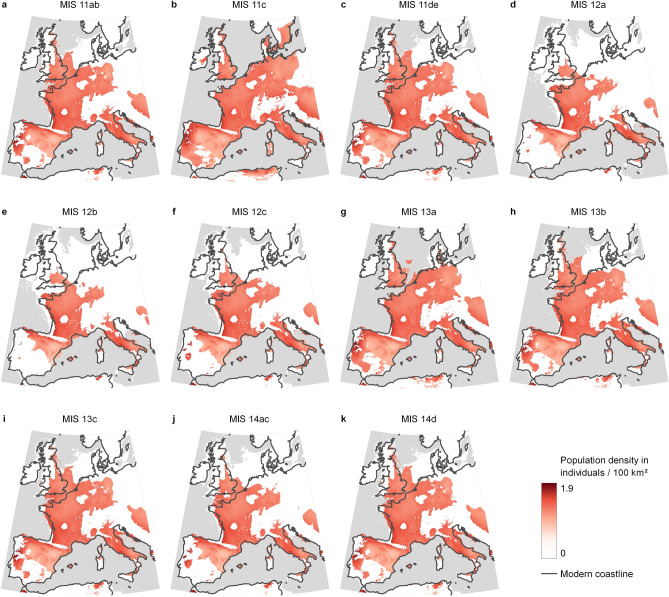
Population density in Western Europe from MIS14 to MIS11. The maps were created in QGIS 3.22 (https://www.qgis.org). in the areas considered suitable for humans based on the SDM. The maps illustrate the lower limit of the estimated interval for population density.

The estimated sustainable population density in Europe is distributed following a consistent pattern throughout the MIS14 to MIS11 periods. A marked West to East gradient is evident, with high values along the Atlantic coast that diminish towards the interior of the continent. This longitudinal gradient is especially relevant in the Iberian Peninsula, where the sustainable population density also shows high variation over time along the Atlantic coast compared with the interior and eastern areas (Fig. 5).

**Figure 6:**
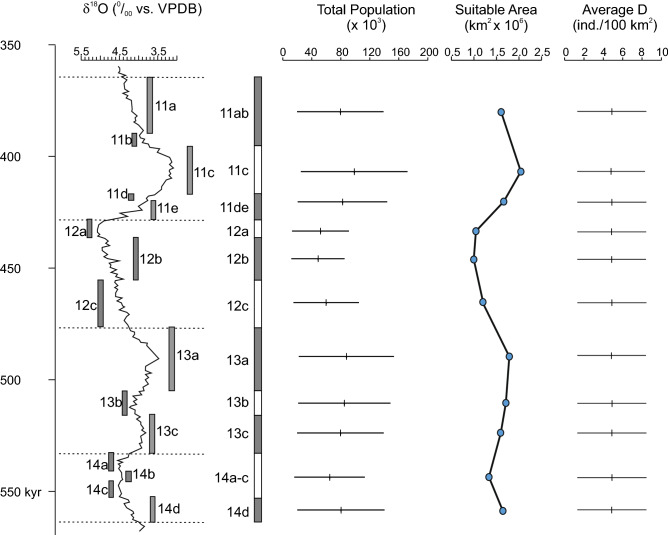
Changes in average population density (D), total population and suitable area in Western Europe during the MIS14-MIS11 period. The LR04 stack of marine benthic foraminiferal δ^18^O^46^ is shown as a proxy for climate variation, indicating the substages defined for the MIS14–MIS11 period ^24^.

The estimated maximum total population along Western Europe shows little variation throughout the MIS14 to MIS11 interval. The maximum value obtained for the MIS11c interval was between 25,000–170,000 individuals while the minimum value for MIS12b was between 13,000–85,000 individuals. This estimate represents a 50% decrease in the potential population at the hardest glacial interval in comparison with the most favourable interglacial interval. Interestingly, the variations in the potential total population of Western Europe were driven by changes in the extent of the area suitable for humans, not by variations in the maximum sustainable density at the local scale (Fig. 6). Indeed, the average population density was constant throughout this period, while the periphery of the suitable area showed marked variation.

## Discussion

It is generally assumed that climate cyclicity severely impacted human distribution in Western Europe during the Middle Pleistocene by restricting the suitable area for humans to the southernmost region of the continent^1,3,8,25,47^. However, the results of the ecological niche model presented here suggest a different scenario. Although most of Britain and large areas north of parallel 50° N were unsuitable for humans during the coldest periods of the MIS14 to MIS11 interval, a core area with favourable conditions for human occupation spread continuously from northern Iberia, Italy, and most of France. Thus, the area suitable for humans during cold periods would have been much larger than normally assumed and would not have been restricted to the Mediterranean peninsulas. Consequently, the populations inhabiting the northwestern area were not entirely disconnected from southern populations as a result of climate shifts, as proposed by some models of human evolution in Europe^1,8,25^. The results of the SDM suggest the existence of a continuous environmental corridor to the east of the Pyrenees during both the cold and warm phases. This corridor would have connected the Iberian and North Western human populations throughout the entire MIS14 to MIS 11 period. In contrast, the Apennine peninsula was disconnected from the rest of the core area during the coldest stages (the entire MIS12 and MIS14 glacial stages). Consequently, human populations in the Apennine peninsula would have been isolated from other populations in Europe during these cold stages, which might have favoured their genetic and cultural divergence from other European populations.

Most scholars agree that Britain was not populated by humans during MIS 12 and MIS 14^8,15,48^. However, the SDM predicts the presence of suitable areas for humans in southern Britain during cold phases. Lithic artefacts have been retrieved from sediments dated to the MIS12 and MIS14 glacial stages in the Bytham River area, but they are usually regarded as reworked items dating back to the former interglacial stage^49^. If this interpretation is correct, and Britain was depopulated during the glacial stages, the SDM might have overestimated the area suitable for humans during those periods. It must be noted, however, that an SDM produces a map of the areas potentially suitable for the target species based on the factors included in the model. In our case, these factors were climate and elevation. Therefore, that area represents an approximation of the fundamental niche^50^ of the Middle Pleistocene European hominins. Although the fundamental niche is generally restricted to biological adaptations, we include here also the potential cultural adaptations of the Middle Pleistocene humans in our use of the term. The realised niche is determined by the overlap of the fundamental niche and the region with an appropriate combination of biotic factors (food resources, competitors, predators, etc.) for the target species^50^. Finally, the region in which a species is actually present is the geographic extent of the realized niche accessible for the species^50^. Thus, even if humans were able to cope with the climatic conditions of Britain during MIS14 and MIS12^23^, other factors, such as the lack of suitable food resources, may have excluded them from that area.

Another unexpected result of the SDM was the exclusion of the Atlantic Coast and the southern half of Iberia from the geographic projection of the fundamental niche of the Middle Pleistocene European hominins for most of the MIS14 to MIS11 period. The Atlantic Coast is significantly more humid, and the southern half is considerably drier and warmer than the rest of the peninsula. There are no other similar climate areas in Western Europe, and there are no sites in that area with a chronology precise enough to be included in the model. Thus, this result should be considered cautiously, as it could change with the discovery of a single site from southern or western Iberia dated to this period.

According to our results, the changes in the total human population that could be sustainable by the environment in Western Europe were largely determined by the variation in the size of the suitable area during the MIS14 to MIS11 period (Fig. 6). The sustainable population density was constant over time and exhibited little spatial variation. Consequently, the effect of changes in population density on the size of the total sustainable population was negligible. The small temporal and spatial variation in population density was a direct consequence of the slight variation in NPP, in time and space, within the area having suitable conditions for humans. It may be argued that our methodology overestimated the amount of resources available to humans, especially in harsh environments, because the Miami model estimates total net primary productivity and only a small amount of that productivity may be directly available to humans. However, our method does not make assumptions about the proportion of NPP actually used by humans. The estimated values of population density are based on the empirical relationship between the population density of recent hunter-gatherers in a given area and the NPP estimated for that area by the Miami model. The proportion of total NPP that can be used by humans varies across different biomes^32^. Thus, it may be expected that the proportion of NPP available to humans in Western Europe was different during glacial and interglacial periods, but this fact does not affect the estimation of population density. The database used to obtain the predictive equations included recent hunter-gatherer populations from a wide sample of biomes, ranging from desert and prairie to taiga and tundra. Thus, it includes the main biomes occurring in Western Europe during the cold and warm phases of the MIS14 to MIS11 periods^51,52^.

Our approach assumes that the population density of Middle Pleistocene hominins may be inferred from ethnographic hunter-gatherer population densities. This assumption may be negatively affected by the biological, cultural and technological differences that exist between the recent hunter-gatherer populations used to derive the empirical relationship between NPP and population density, and the Lower Palaeolithic hunter-gatherers. It has been proposed that Neanderthals lived in smaller groups than recent hunter-gatherers based on genetic data^22^. Differences in social organization, derived from differences in their cognitive capacities, might force Middle Pleistocene humans to live also in small groups and small social networks. This would have an impact on their population density. It has been proposed that group size is related to brain size in primates^53^, but see also^54^, and the brain size of the Middle Pleistocene European hominins was similar to, but smaller than, the brain size of *Homo sapiens*^55^. Moreover, recent hunter-gatherers use sophisticated hunting weaponry and a set of food conservation techniques not available to Lower Palaeolithic hunter-gatherers. These differences likely make recent hunter-gatherers more efficient than Lower Palaeolithic hunter-gatherers in extracting and processing resources, allowing them to co-opt a larger proportion of NPP and achieve higher population densities than Lower Palaeolithic hunter-gatherers in an equivalent environment. This is a major factor that may have inflated the estimated population density values for the Middle Pleistocene populations. However, the large similarities in body size, diet, and physiology between Middle Pleistocene hominins and *H. sapiens* support the validity of the aforementioned assumption. Therefore, taking the estimated values of population density with caution, only the lower limit of the estimated interval for population density will be considered in the following discussion.

Following this conservative approach, the total sustainable population in Western Europe during the MIS14–MIS11 interval would have oscillated between 13,000 and 25,000 individuals. Models of human evolution in Europe during the Middle Pleistocene often estimate a census population five to ten times lower^8^, giving an effective breeding population as low as 600–1,000 individuals in full glacial times and 1,200–2,000 individuals during warm periods^8^. There is strong theoretical and empirical evidence suggesting that the optimal size for a hunter-gatherer group or band is 25 individuals^41^, although the validity of the multi-scalar organization pattern commonly assumed for hunter-gatherer societies has recently been questioned^54^. Assuming an average size of 25 individuals for a Lower Palaeolithic hunter-gatherer band, it has been estimated that there may have been 50–100 such bands in Europe during full glacial periods and 120–200 bands during interstadials^8^. According to the widespread assumption that the European population was confined to refugia in the Iberian, Apennine, and Balkan Peninsula during the glacial stages^1,3,8,25^, only 20–30 such groups would have been isolated within each peninsula. In this scenario, the probability of interbreeding between groups of the same peninsula would be very low, and the genetic flow between populations in different peninsulas would have been impossible for several millennia. Moreover, assuming that the total census population was 1,500–2,000 individuals, with only 40% being of reproductive age^8^; that interbreeding between populations from different peninsulas was not possible; and, for the sake of simplicity, that the total population was equally distributed among the three areas; the effective breeding size of each isolated population would have been only 200–333 individuals. Under these assumptions, the census population would be 500–833 for each peninsula, which is much lower than the minimum viable population size for any organism, which exceeds a few thousand individuals^56^. In contrast, under the new scenario described here, the number of bands in Western Europe could have been as many as 520 in full glacial periods and up to 1,000 during warm periods. Moreover, the Iberian bands were connected to the north of the Pyrenees during MIS12 and MIS14, allowing genetic flow between them and the bands in the north. Only the population from the Apennine peninsula would have been isolated from the others; however, according to the minimum sustainable population density estimated here for that area during MIS14 and MIS12, this population could have exceeded 2,000 individuals. A population of that size isolated in the Apennine peninsula over several millennia could be viable, although it would also have a high risk of extinction. In contrast, the main metapopulation inhabiting France and Iberia could attain a size far above the threshold for a viable population during the entire MIS14 to MIS11 period.

The estimated values of the total population reported here represent the maximum number of individuals that the Western European ecosystems could sustain during the MIS14 to MIS11 periods. These estimations represent an ecological baseline from which to think about Middle Palaeolithic population history in Western Europe. Other factors not included in our approach, like the cultural differences among groups, biological singularities of the Middle Pleistocene hominins, diseases or stochastic events, likely also played a role in determining the actual size of the Middle Pleistocene populations. Therefore, our results should be considered the maximum thresholds, and it must be acknowledged that the actual population size may have been lower. However, these numbers, combined with the geographic projection of the fundamental niche of Middle Pleistocene humans produced by the SDM, set up a new theoretical scenario for models and hypotheses aimed at explaining cultural and biological changes in Western Europe during the critical period of human evolution known as the Middle Pleistocene.

## Methods

### Occurrence data

This study used a published dataset of archaeological assemblages correlated with the interval from MIS14 to MIS11^23^ compiled from published literature. The dataset included archaeological assemblages from the European continent and the British Islands west of the meridian 14^o^E, along with the entire Apennine peninsula. No latitudinal limits were established within the European continent for sites to be included in the dataset. Each archaeological assemblage was correlated to the marine isotope curve^24^ according to information provided by the original sources on the biostratigraphy, chronostratigraphy and radiometric dates for the site^23^. The dataset includes 68 archaeological assemblages from 46 sites, but because of relatively imprecise dating methods available for this period, only 33 assemblages correlated to an interval of the marine isotope curve (Supplementary Table S1). The other 35 assemblages could not be correlated with certainty to an interval within a stage^23^.

### Palaeoenvironmental data

Palaeoclimate data for the MIS14–MIS11 period were obtained from the Oscillayers palaeoclimatic database^26,27^. Oscillayers includes interpolated estimates for 19 bioclimatic variables (BIO1 to BIO19) at high spatial (2.5 arc-min) and temporal (10 ka time periods) resolution and covers the period from 5.4 million years ago to the present^26^. The set of 19 bioclimatic variables provided in Oscillayers is the same as that defined in the BIOCLIM model^57^: these variables are commonly used in ecological niche modelling analyses^58^. Because the duration of intervals varied from 10 to 30 ka, it was necessary in some cases to aggregate several Oscillayers time slices (Supplementary Table S2). When an interval overlapped the time period represented by two or more Oscillayer time slices, the values of the bioclimatic variables were obtained as the average ^23^. A map of NPP in grams of dry matter/m^2^·yr was estimated for each interval using the Miami model^59^ with the mean annual temperature (BIO1) and total annual precipitation (BIO12) obtained from the Oscillayers dataset.

All geodatasets in this study were transformed to the Lambert azimuth equal-area projection based on the ETRS89 ellipsoid. With a projection centre of Lon =  + 52° and Lat =  + 10 ^o^, as defined in EPSG:3035, this allows high accuracy area measurements.

### Ecological niche modelling

We chose to describe and infer the human niche across diverse climate regions during multiple warm and cold phases. For this, we selected statistically sound covariates to describe the environment and generated a sample accounting for the spatial surroundings of the sites. We performed computational tests on species SDMs using these data to assess the range of hominin habitable zones.

Regarding environmental factors, we started with 19 bioclimatic variables from the Oscillayer dataset and a digital elevation model. Elevation is usually deprecated as a proxy describing the landscape^60^. However, it was included here to evaluate if adaptation to height might play a role in the distribution of the target hominin populations. More sophisticated topographic variables derived from high-resolution DEMs are often involved in studies targeted at site prediction, where they exert strong predictive power. The distribution of early archaeological sites is often related to certain types of geology and topography (e.g. limestone caves, gorges, undercuts) due to site preservation factors. However, the aim here is not the prediction of sites but producing an approximation to the human niche. Therefore, we excluded the complex and biased factor ‘landscape’ from our study. Because the Maximum Entropy (MaxEnt) algorithm provides functions for expanding the covariates (i.e. the input environmental variables) into complex feature classes, it is recommended to reduce the number of variables in advance to prevent undesirable effects caused by correlation and multicollinearity^61,62^. A common approach to reduce dimensionality is to project the variables onto principal components^63,64^. However, this comes at the price of a reduced SDM interpretability or highly noncausal relationships^65^. Instead, we selected meaningful covariates through thresholds. Therefore, we calculated a pairwise correlation matrix of all variables with the R package “Hmisc” v.4.5^66^ and systematically excluded those that were correlated with others using a Pearson or Spearman correlation coefficient of |r|> 0.9. Where multiple correlated variables existed, we retained the more general variable (e.g. “Annual Mean Temperature” over “Mean Temperature of Coldest Quarter”). We further assessed the issue of multicollinearity between variables by calculating R^2^, Tolerance, and Variance Inflation Factor (VIF) with the package “fuzzySim” v.3.0^67^. These indicators describe the linear dependence of one variable on multiple other variables (Supplementary Table S3 and Supplementary Figure S3). Since we found concerning amounts of collinearity, we iteratively reduced the variables with the highest VIF until only those variables with a threshold VIF < 5 remained. Recommended thresholds lie at VIF < 10, VIF < 5, or VIF < 3^68,69^. Through this selection process, the candidate variables elevation, BIO1, BIO2, BIO7, BIO12 and BIO15 were chosen as suitable covariates for further modelling.

The locations of archaeological finds are often related to specific geologic and taphonomic conditions and do not necessarily reflect the environmental variability of the landscape that allowed early humans to survive. However, these surroundings are elementary for hunting and gathering lifestyles and need to be addressed in an SDM. Therefore, we expanded the occurrence site locations by including their geographical surroundings within a buffer radius of 10 km from the sample. This radius corresponds to maximal one-way distances of daily foraging trips of modern hunter-gatherers^41^ and resulted in a total sample size of 664 points around 30 sites. Where sites were occupied multiple times and assemblages lay in different substages, these were treated as individual sites with their respective environmental conditions. We accounted for spatial autocorrelation by creating a grid that allowed only one sample per raster cell. Furthermore, only onshore cells were considered, based on the palaeo sea-level estimates within the Oscillayer dataset, which rely on the sea-level reconstructions and the ETOPO1 elevation model^27,70^.

Because our archaeological dataset is based on occurrences and lacks confirmed absence data, we chose the MaxEnt algorithm, which is considered a powerful tool for SDM with presence and background data^71–73^. Our analyses were conducted with the software ENMeval v.2.0.1^42^, which relies on MaxEnt v.3.4.4^74^ and is available in the statistical software R v.4.0.5^75^. The SDM was calibrated by performing tests with the following alternate configurations:

- The feature space was enhanced by applying six functions (L, LQ, LQH, LQP, LQHP, and LQHPT with L = linear, Q = quadratic, H = hinge, P = product, T = threshold) to the initial covariates.
- We tested eight regularisation multipliers (0.2, 0.4, 0.6, 0.8, 1.5, 2, 3, and 4) in addition to the default value of 1.0.
- Because MaxEnt is capable of penalizing covariates and because of our rigid preselection procedure, we did not perform additional tests with varying covariate sets.

Each of these experiments was tested with the same 10,000 background points, initial covariates and sample data. A sensitivity analysis showed that the distribution of the sampled values can be considered stable when the sample exceeds the size of n > 1,000 (Figure S4a). The models were run with 500 iterations and a k-fold subsampling strategy to ensure that sites and their surroundings were treated as a whole. In earlier attempts, we noticed that completely random subsets resulted in heavily over-fitted models; this was attributed to spatial autocorrelation from our sampling design that characterized single sites by multiple points.

**Figure 7:**
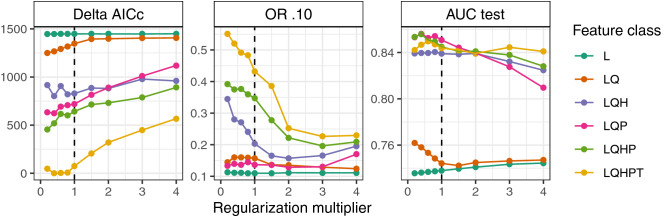
Performance of model candidates evaluated with the indicators Akaike Information Criterion for small samples (AICc), Omission Rate of the testing points at 10% training threshold and Area Under ROC Curve (AUC). The models varied in using feature classes (colour) and the regularisation multiplier (x-axis). The dashed vertical line indicates the default model with a default regularisation of 1.0.

We evaluated these models based on three indicators (Fig. 7), each computed with ENMeval: the Akaike information criterion for small sample sizes relative to the “best” performing model (∆AICc), the average Omission Rate of the 10%-percentile of presence points (OR.10), and the area under the receiver-operator-curve (AUC) of the test subsample^76^. The model performance results showed, that the feature set choice (indicated by colour) majorly affected the model quality. Particularly, those models that outperformed in one indicator performed poorly in others. For example, the feature set LQHPT performed best in AICc but worst in OR.10, whereas feature set L had the best performance in OR.10 but poor values regarding AICc and AUC. Therefore, we chose a compromise model, with a feature set LQP and a regularisation multiplier of 0.4. This model combined an ∆AICc = 624 (AICc = 10,504) lying in the midfield, a good OR.10 = 0.14, and a high AUC = 0.86.

We projected this model onto the environmental data of the 11 time periods and thus produced continuous surfaces with log transformations (1 = most suitable, 0 = least suitable), indicating suitability for human occurrences. These were further binarised to create a mask of the human habitat, based on a threshold of suitability = 0.118, corresponding to the 5%-percentile of predicted values at observed sites (see also^28^).

The 33 archaeological assemblages not correlated with certainty to an interval within a stage were used for further validation of the model (Supplementary Table S4).

### Estimation of population density

A dataset of recent hunter-gatherer populations compiled by L.R. Binford^32^ and data on NPP^31^ were used to obtain a predictive equation of the maximum sustainable hunter-gatherer population density in individuals/100 km^2^ from NPP in grams of dry matter /m^2^·yr. The data on recent NPP are based on estimations from the Miami model^21,59^. This source was preferred to satellite-based values for two reasons. First, climate-based NPP estimates avoid the potential problems caused by recent human appropriations of NPP^21^, and second, we used the Miami model to estimate NPP in the past, making our estimates of NPP comparable through time. Latitude, longitude, population density, and percentage of fishing, hunting, and gathering in the diet of 215 Holarctic hunter-gatherer groups living above the parallel 30° N were obtained from Binford’s dataset^32^ (Supplementary Table S5). The population density was obtained by dividing the total population of the ethnic group by the total area occupied^32^. For the hunter-gatherer groups with additional population density estimates available^41^, we used the average of the two estimates. As highlighted by other authors^21,32^, the sample of recent hunter-gatherers in Binford’s dataset is geographically biased but is not biased concerning niche space or habitat. Hunter-gatherer societies considered dependent on trade with non-forager societies for their subsistence at the time of documentation^32^ were excluded. K-means clustering was used to classify the hunter-gatherer groups according to their procurement strategy using the percentage of hunting, fishing and gathering in their diet as input data (Supplementary Table S6). This analysis confirmed the validity of the classification of hunter-gatherer groups as ‘hunters’, ‘fishers’ and ‘gatherers’ provided by the original source^32^ based on their most frequent activity. However, because our results and the original source differed in the assignment of some hunter-gatherer groups to a procurement strategy group^32^, we retained the classification produced by k-means clustering in the subsequent analyses.

Population density and NPP were transformed to the logarithm of base 10 (LOGD and LOGNPP, respectively), and ordinary least square regression was used to fit a function to the relationship between LOGD and LOGNPP for the entire sample as well as for each separate procurement strategy group. One case was excluded from the analyses (Monachi-southern) after being identified as an outlier (out of the 95% prediction interval for the cases). Horseback hunting has been suggested to allow some hunter-gatherer groups to occur at higher densities than on-foot hunters at a given value of NPP^21^. Thus, we tested the effect of removing mounted hunters from the sample used to build the regression model. Regression equations were fitted to the upper and lower limits of the distributions of ‘Hunters’ and ‘Gatherers’^33,77^. The sample was divided into bins of 0.1 LOGNPP and the regression equations were fitted to the maximum and minimum population density for each bin. The lower and upper limit equations for ‘Hunters’ were applied to the NPP map for each time interval to obtain maps that estimated the maximum and minimum population densities at each cell. The maximum and minimum total populations in Western Europe for each time interval were estimated as:

$$P=\overline{D }\times A$$

where P is the total population, $\overline{D }$ is the average population density (individuals/100 km^2^) within the range predicted for the substage and A is the size of that range in km^2^. Two P estimates were obtained for each time interval, the first from the maximum population density estimate and the second from the minimum population density estimate. K-means clustering and regression analyses were carried out using the StatSoft Statistica 13.

## Supplementary Information

Supplementary Information 1.

Supplementary Information 2.

## Data Availability

All maps, R scripts and the clipped and re-projected Oscillayer datasets generated during the current study are available in ^78^. All other data generated or analysed during this study are included in this published article and its Supplementary Information files.

## References

[1] Dennell R (2003). Dispersal and colonisation, long and short chronologies: how continuous is the Early Pleistocene record for hominids outside East Africa?. J. Hum. Evol..

[2] Moncel M-H (2020). Early Levallois core technology between Marine Isotope Stage 12 and 9 in Western Europe. J. Hum. Evol..

[3] Moncel M-H (2018). Linking environmental changes with human occupations between 900 and 400 ka in Western Europe. Quatern. Int..

[4] Meyer M (2016). Nuclear DNA sequences from the Middle Pleistocene Sima de los Huesos hominins. Nature.

[5] Meyer M (2014). A mitochondrial genome sequence of a hominin from Sima de los Huesos. Nature.

[6] Rightmire GP (2008). *Homo* in the Middle Pleistocene: Hypodigms, variation, and species recognition. Evolut. Anthropol. Issues News Rev..

[7] Stringer CB (2012). The Status of *Homo heidelbergensis* (Schoetensack 1908). Evol. Anthropol..

[8] Dennell RW, Martinón-Torres M, Bermúdez de Castro JM (2011). Hominin variability, climatic instability and population demography in Middle Pleistocene Europe. Quat. Sci. Rev..

[9] Galway-Witham J, Cole J, Stringer C (2019). Aspects of human physical and behavioural evolution during the last 1 million years. J. Quat. Sci..

[10] Powell A, Shennan S, Thomas MG (2009). Late Pleistocene Demography and the Appearance of Modern Human Behavior. Science.

[11] Vaesen K, Collard M, Cosgrove R, Roebroeks W (2016). Population size does not explain past changes in cultural complexity. Proc. Natl. Acad. Sci..

[12] Henrich J (2004). Demography and cultural evolution: how adaptive cultural processes can produce maladaptive losses: the Tasmanian case. Am. Antiq..

[13] Cavalli-Sforza L, Barrai I, Edwards AWF (2004). Analysis of human evolution under random genetic drift. Symp. Quant. Biol..

[14] Boaz NT (1979). Early hominid population densities: new estimates. Science.

[15] Ashton N, Davis R (2021). Cultural mosaics, social structure, and identity: the Acheulean threshold in Europe. J. Hum. Evol..

[16] Hayden B (2012). Neandertal social structure?. Oxf. J. Archaeol..

[17] Bocquet-Appel J-P, Demars P-Y, Noiret L, Dobrowsky D (2005). Estimates of Upper Paleolithic meta-population size in Europe from archaeological data. J. Archaeol. Sci..

[18] Maier A (2016). Demographic estimates of hunter–gatherers during the Last Glacial Maximum in Europe against the background of palaeoenvironmental data. Quatern. Int..

[19] Gautney JR, Holliday TW (2015). New estimations of habitable land area and human population size at the Last Glacial Maximum. J. Archaeol. Sci..

[20] Rodríguez-Gómez G, Rodríguez J, Martín-González JA, Goikoetxea I, Mateos A (2013). Modeling trophic resource availability for the first human settlers of Europe: the case of Atapuerca TD6. J. Hum. Evol..

[21] Tallavaara M, Luoto M, Korhonen N, Järvinen H, Seppä H (2015). Human population dynamics in Europe over the Last Glacial Maximum. Proc. Natl. Acad. Sci..

[22] Sánchez-Quinto F, Lalueza-Fox C (2015). Almost 20 years of Neanderthal palaeogenetics: adaptation, admixture, diversity, demography and extinction. Philosophical Trans. Royal Soc. B Biol. Sci..

[23] Rodríguez J, Willmes C, Mateos A (2021). Shivering in the Pleistocene. Human adaptations to cold exposure in Western Europe from MIS 14 to MIS 11. J. Hum. Evol..

[24] Railsback LB, Gibbard PL, Head MJ, Voarintsoa NRG, Toucanne S (2015). An optimized scheme of lettered marine isotope substages for the last 1.0 million years, and the climatostratigraphic nature of isotope stages and substages. Quatern. Sci. Rev..

[25] MacDonald K, Martinón-Torres M, Dennell RW, Bermúdez de Castro JM (2012). Discontinuity in the record for hominin occupation in south-western Europe: implications for occupation of the middle latitudes of Europe. Quatern. Int.

[26] Gamisch A (2019). Oscillayers: A dataset for the study of climatic oscillations over Plio-Pleistocene time-scales at high spatial-temporal resolution. Glob. Ecol. Biogeogr..

[27] Gamisch, A. Oscillayers: A dataset for the study of climatic oscillations over Plio-Pleistocene time-scales at high spatial-temporal resolution. 10.5061/dryad.27f8s90 (Dryad, 2019). 10.1111/geb.12979 PMC6853231 31762691

[28] Banks WE (2021). An ecological niche shift for Neanderthal populations in Western Europe 70,000 years ago. Sci. Rep..

[29] Banks WE, d'Errico F, Zilhão J (2013). Human–climate interaction during the Early Upper Paleolithic: testing the hypothesis of an adaptive shift between the Proto-Aurignacian and the Early Aurignacian. J. Hum. Evol..

[30] Soberón J, Nakamura M (2009). Niches and distributional areas: Concepts, methods, and assumptions. Proc. Natl. Acad. Sci..

[31] Tallavaara M, Eronen JT, Luoto M (2018). Productivity, biodiversity, and pathogens influence the global hunter-gatherer population density. Proc. Natl. Acad. Sci..

[32] Binford LR (2001). Constructing frames of reference: an analytical method for archaeological theory building using ethnographic and environmental data set.

[33] Hamilton MJ, Milne BT, Walker RS, Brown JH (2007). Nonlinear scaling of space use in human hunter–gatherers. Proc. Natl. Acad. Sci..

[34] Coe MJ, Cumming DH, Phillipson J (1976). Biomass and production of large African herbivores in relation to rainfall and primary production. Oecologia.

[35] Hatton IA (2015). The predator-prey power law: biomass scaling across terrestrial and aquatic biomes. Science.

[36] Carbone C, Gittleman JL (2002). A common rule for the scaling of carnivore density. Science.

[37] Braun DR (2010). Early hominin diet included diverse terrestrial and aquatic animals 1.95 Ma in East Turkana, Kenya. Proc Natl Acad Sci.

[38] Marlowe FW (2005). Hunter-gatherers and human evolution. Evolut. Anthropol. Issues News Rev..

[39] Steele T (2010). A unique hominin menu dated to 1.95 million years ago. Proc. Natl Acad Sci United States of Am.

[40] Conard NJ (2015). Excavations at Schöningen and paradigm shifts in human evolution. J. Hum. Evol..

[41] Kelly RL (2013). The lifeways of hunter-gatherers: the foraging spectrum.

[42] Muscarella R (2014). ENMeval: An R package for conducting spatially independent evaluations and estimating optimal model complexity for Maxent ecological niche models. Methods Ecol. Evol..

[43] Radosavljevic A, Anderson RP (2014). Making better Maxent models of species distributions: complexity, overfitting and evaluation. J. Biogeogr..

[44] Morales NS, Fernández I, Baca-González V (2017). MaxEnt’s parameter configuration and small samples: are we paying attention to recommendations? A systematic review. PeerJ.

[45] Anderson RP, Gonzalez I (2011). Species-specific tuning increases robustness to sampling bias in models of species distributions: an implementation with Maxent. Ecol. Model..

[46] Lisiecki L, Raymo M (2005). A Pliocene-Pleistocene stack of 57 globally distributed benthic ^18^O records. Paleoceanography.

[47] Carrión JS, Rose J, Stringer CB (2011). Early human evolution in the western Palaearctic: ecological scenarios. Quat. Sci. Rev..

[48] Davis R, Ashton N (2019). Landscapes, environments and societies: the development of culture in Lower Palaeolithic Europe. J. Anthropol. Archaeol..

[49] Davis R, Ashton N, Hatch M, Hoare PG, Lewis SG (2021). Palaeolithic archaeology of the Bytham River: human occupation of Britain during the early Middle Pleistocene and its European context. J. Quat. Sci..

[50] Soberón J, Peterson A (2005). Interpretation of models of fundamental ecological niches and species’ distributional areas. Biodivers. Inform..

[51] Kahlke R-D (2011). Western Palaearctic palaeoenvironmental conditions during the Early and early Middle Pleistocene inferred from large mammal communities, and implications for hominin dispersal in Europe. Quat. Sci. Rev..

[52] Hosfield R (2020). The earliest Europeans a year in the life.

[53] Dunbar RIM (1992). Neocortex size as a constraint on group size in primates. J. Hum. Evol..

[54] Bird DW, Bird RB, Codding BF, Zeanah DW (2019). Variability in the organization and size of hunter-gatherer groups: foragers do not live in small-scale societies. J. Hum. Evol..

[55] Arsuaga JL (2014). Neandertal roots: Cranial and chronological evidence from Sima de los Huesos. Science.

[56] Traill LW, Bradshaw RHW, Brook BW (2007). Minimum viable population size: a meta-analysis of 30 years of published estimates. Biol. Cons..

[57] Booth TH, Nix HA, Busby JR, Hutchinson MF (2014). BIOCLIM: the first species distribution modelling package, its early applications and relevance to most current MaxEnt studies. Divers. Distrib..

[58] Hijmans RJ, Cameron SE, Parra JL, Jones PG, Jarvis A (2005). Very high resolution interpolated climate surfaces for global land areas. Int. J. Climatol..

[59] Lieth HFH (1973). Primary production: terrestrial ecosystems. Hum. Ecol..

[60] Guisan A, Zimmermann NE (2000). Predictive habitat distribution models in ecology. Ecol. Model..

[61] Merow C, Smith MJ, Silander JA (2013). A practical guide to MaxEnt for modeling species’ distributions: what it does, and why inputs and settings matter. Ecography.

[62] Braunisch V (2013). Selecting from correlated climate variables: a major source of uncertainty for predicting species distributions under climate change. Ecography.

[63] De Marco PJ, Nóbrega CC (2018). Evaluating collinearity effects on species distribution models: an approach based on virtual species simulation. PLoS ONE.

[64] Dormann CF (2013). Collinearity: a review of methods to deal with it and a simulation study evaluating their performance. Ecography.

[65] Fourcade Y, Besnard AG, Secondi J (2018). Paintings predict the distribution of species, or the challenge of selecting environmental predictors and evaluation statistics. Glob. Ecol. Biogeogr..

[66] Harell Jr., F. E. & with contributions from Charles Dupont and many others. Hmisc: Harrell Miscellaneous (2021).

[67] Barbosa AM (2015). fuzzySim: applying fuzzy logic to binary similarity indices in ecology. Methods Ecol. Evol..

[68] Zuur AF, Ieno EN, Elphick CS (2010). A protocol for data exploration to avoid common statistical problems. Methods Ecol. Evol..

[69] James, G., Witten, D., Hastie, T. & Tibshirani, R. *An Introduction to Statistical Learning with Appllication in R*. 1 edn, (Springer, 2013).

[70] Amante, C. & Eakins, B. ETOPO1 1 Arc-Minute Global Relief Model: procedures, data sources and analysis. 10.7289/V5C8276M (2009).

[71] Elith J (2006). Novel methods improve prediction of species’ distributions from occurrence data. Ecography.

[72] Tsoar A, Allouche O, Steinitz O, Rotem D, Kadmon R (2007). A comparative evaluation of presence-only methods for modelling species distribution. Divers. Distrib..

[73] Phillips SJ, Dudík M (2008). Modeling of species distributions with Maxent: new extensions and a comprehensive evaluation. Ecography.

[74] Phillips SJ, Anderson RP, Dudík M, Schapire RE, Blair ME (2017). Opening the black box: an open-source release of Maxent. Ecography.

[75] 755026R: A Language and Environment for STATISTICAL Computing (R Foundation for Statistical Computing, Vienna, Austria, 2021).

[76] Warren DL, Seifert SN (2011). Ecological niche modeling in Maxent: the importance of model complexity and the performance of model selection criteria. Ecol. Appl..

[77] Kelt D, Vuren D (2001). The ecology and macroecology of mammalian home range area. Am. Nat..

[78] Rodríguez, J., Sommer, C., Willmes, C. & Mateos, A. Data and code for "Sustainable Human Population Density in Western Europe between 560.000 and 360.000 years ago" 10.5281/zenodo.6045917 (2022). 10.1038/s41598-022-10642-w PMC9051054 35484382

