# Supplementary material for: Sustainable human population density in Western Europe between 560.000 and 360.000 years ago
Source: Sci Rep. 2022 Apr 28;12:6907. doi: 10.1038/s41598-022-10642-w (PMC9051054; doi:10.1038/s41598-022-10642-w)
Supplement: Supplementary file 2 — Supplementary Information 2. [file 41598_2022_10642_MOESM2_ESM.pdf]

Supplementary Information for

Sustainable Human Population Density in Western Europe between 560.000 and 360.000 years ago

Jesús Rodríguez[1]*

Christian Willmes[2]

Christian Sommer[3]

Ana Mateos[1]

[1] Centro Nacional de Investigación sobre la Evolución Humana (CENIEH), Paseo Sierra de Atapuerca 3, 09002, Burgos, Spain.

[2] Institute of Geography, University of Cologne, 50923 Cologne, Germany.

[3] The Role of Culture in Early Expansions of Humans, Heidelberg Academy of Sciences and Humanities at the University of Tübingen, Research Area Geography, Rümelinstr. 19-23. 72070, Tübingen, Germany

*Corresponding author. jesus.rodriguez@cenieh.es

Supplementary Figures
Supplementary Tables
Supplementary References

Table S1. Archaeological sites from MIS14 to MIS11 used to compute and validate the ecological niche model for hominins. This table is an Excel spreadsheet contained in an individual tab in the Excel file available for download in 'Supplementary Information.'

Locality	Unit	Longitude	Latitude	MIS Correlation	Ref.
Cagny-La-Garenne II	Levels I & H	2.34	49.87	MIS 11	1
Ariendorf	1	7.28	50.53	MIS 11	2,3
Schoeningen	Scho 12	11.03	52.18	MIS 11	4-7
Bilzingsleben II	II	11.07	51.28	MIS 11	2,4,8
Atapuerca Gran Dolina	TD10-3	-3.52	42.35	MIS 11	9
Malagrotta	-	12.33	41.88	MIS 11	10
Terra Amata	C1a	7.25	43.7	MIS 11	11
Terra Amata	C1b	7.25	43.7	MIS 11	11,12
Ambrona	AS1-4	-2.508	41.166	MIS 11	13,14
Beeches Pit	6	0.64	52.3	MIS 11	15
Beeches Pit	5	0.64	52.3	MIS 11	15
Beeches Pit	7	0.64	52.3	MIS 11	15
Le Grande Vallée	U5a - U5e	0.46	46.68	MIS 11	16
Menez-Dregan I	Layer 7	-4.49	48.5	MIS 11	17-19
Aldéne	TU II	2.7	43.35	MIS 11	20
Schladebach/Wallendorf	-	12.09	51.33	MIS 11	21
Aridos 1	-	-3.53	40.15	MIS 11a	22,23
Swanscombe	Upper Loam	0.32	51.42	MIS 11a	24
Aridos 2	-	-3.53	40.15	MIS 11a	22,23
Isoletta	GA6Z	13.57	41.53	MIS 11a	25
La Cansaladeta	A, B, C , D	1.18	41.31	MIS 11a	26
Hoxne	A	1.19	52.35	MIS 11a	24,27
Hoxne	B	1.19	52.35	MIS 11a	24,27
Atapuerca Gran Dolina	TD10-1	-3.52	42.35	MIS 11a-b	9
Fontana Ranuccio	FR 4	13.35	41.63	MIS 11a-b	25,28,29
Ambrona	AS1	-2.508	41.166	MIS 11a-b	13,14
Ambrona	AS2	-2.508	41.166	MIS 11a-b	13,14
Guado San Nicola	SU A*B, SU B, SU B*C, SU C and SU C	14.16	41.52	MIS 11a-b	30
Cava Pompi	Level 5	13.44	41.56	MIS 11a-b	25
Saint-Acheul	Tufa	2.31	49.87	MIS 11a-b	31
Uichteritz	Middle Gravel	11.91	51.21	MIS 11a-c	32
La Cansaladeta	I - J	1.18	41.31	MIS 11b	15
Barnham	5c	0.75	52.37	MIS 11c	24,33
Swanscombe	Lower loam	0.32	51.42	MIS 11c	24,33
Atapuerca Gran Dolina	TD10-2	-3.52	42.35	MIS 11c	9

Locality	Unit	Longitude	Latitude	MIS Correlation	Ref.
La Celle-sous-Moret	Tufa	2.85	48.38	MIS 11c	[1,34]
Clacton	Freshwater beds	1.09	51	MIS 11c	[24]
Lademagne	102 m a.s.l.	13.48	41.53	MIS 11c	[25]
Beeches Pit	3b	0.64	52.3	MIS 11c	[15]
Aroeira	layer X	-8.62	39.51	MIS 11c	[35,36]
La Cansaladeta	K	1.18	41.31	MIS 11c	[15]
Londigny	IIBt horizon	0.15	46.08	MIS 11c	[37]
Castel di Guido	-	12.28	41.9	MIS 11d-e	[10,28]
Kärlich	Level H	7.47	50.47	MIS 12	[4,38]
Boxgrove	6b, 8a, 8b,	-0.72	50.85	MIS 12	[39]
Atapuerca Gran Dolina	TD10-4	-3.52	42.35	MIS 12	[9]
Caune de l'Arago	CM_III	2.66	42.79	MIS 12	[40-42]
Cimitero di Atella	L	15.66	40.88	MIS 12	[43,44]
Aldène	TU I	2.7	43.35	MIS 12	[20]
Atapuerca Sima de los Huesos	LU 6	-3.52	42.35	MIS 12	[45-47]
Cagny-La-Garenne	-	2.34	49.86	MIS 12a-b	[1]
Cagny-Cimetière	Basal gravels	2.33	49.86	MIS 12a-b	[34,48]
Valle Giumentina	VV1 LABM	14.02	42.18	MIS 12b-c	[49,50]
Attenfeld	-	11.2	48.78	MIS 12c	[4]
High Lodge	Bed E	0.56	52.35	MIS 12c	[51,52]
Cueva de Bolomor	Fase I	-0.24	39.06	MIS 13	[53,54]
Boxgrove	4b	-0.72	50.85	MIS 13	[39,55]
Boxgrove	5a	-0.72	50.85	MIS 13	[39,55]
Boxgrove	4c	-0.72	50.85	MIS 13	[39,55]
Caune de l'Arago	CM_II	2.66	42.79	MIS 13	[40-42]
Valdoe	Slindon sands	-0.72	50.94	MIS 13	[56,57]
Happisburgh 1	Organic mud and grey sands	1.54	52.82	MIS 13	[51,52,58]
High Lodge	Bed C	0.56	52.35	MIS 13	[51,52,58]
Waverly Wood	-	-1.46	52.34	MIS 13	[59]
Ficoncella	FIC 1	11.88	42.22	MIS 13a	[60]
Valle Giumentina	VV1 LAN	14.02	42.18	MIS 13c	[49,50]
Caune de l'Arago	CM_I	2.66	42.79	MIS 14	[40-42]
Rue du Manège	-	2.28	49.88	MIS 14a-c	[61]

Table S2. Correspondence between time intervals based on the substages of the Marine Isotope Stratigraphy[62] and the Oscillayers time slices[63].

Time interval (MIS substage)	Chronology	Oscillayers time slices
MIS 11ab	370–390 ka	t37, t38
MIS 11c	390-420 ka	t39,t40, t41
MIS 11de	420-430 ka	t42,
MIS 12a	430-440 ka	t43
MIS 12b	440-460 ka	t44, t45
MIS 12c	460-480 ka	t46, t47
MIS 13a	480-500 ka	t48, t49, t50
MIS 13b	500-510 ka	t51
MIS 13c	520-530 ka	t52, t53
MIS 14a-c	530-550 ka	t54, t55
MIS 14d	550-560 ka	t56

Table S3. Collinearity measures for feature reduction. The table shows the reduction of collinearity between environmental variables and, hence, the selection of the most informative variables over several steps. The coefficient of determination of a linear regression between a variable and all other variables (R^2), the tolerance ($TOL=1-R^2$), and the Variance Inflation Factor (VIF) are reported for each step. In step 1, we excluded all variables having a pairwise correlation of $|r|>0.9$ with another variable from the dataset (see figure S3). In step 2, we iteratively excluded the variable with the highest VIF until all variables reached a threshold of VIF<5.

	All variables			Step 1: $\|r\|<0.9$			Step 2: VIF<5		
**Variable**	**R^2**	**TOL**	**VIF**	**R^2**	**TOL**	**VIF**	**R^2**	**TOL**	**VIF**
bio1	0.999	0.001	863.2	0.730	0.270	3.7	0.677	0.323	3.1
bio2	0.989	0.011	88.4	0.977	0.023	43.1	0.658	0.342	2.9
bio3	0.983	0.017	57.6	0.978	0.022	46.5			
bio4	0.999	0.001	1181.5						
bio5	1.000	0.000	33814.7						
bio6	1.000	0.000	52181.3						
bio7	1.000	0.000	14762.6	0.972	0.028	35.4	0.636	0.364	2.7
bio8	0.853	0.147	6.8						
bio9	0.964	0.036	27.8						
bio10	1.000	0.000	2713.0						
bio11	1.000	0.000	5199.4						
bio12	0.993	0.007	141.8	0.990	0.010	102.3	0.347	0.653	1.5
bio13	0.991	0.009	112.9						
bio14	0.989	0.011	87.2						
bio15	0.935	0.065	15.3	0.903	0.097	10.3	0.185	0.815	1.2
bio16	0.996	0.004	236.5	0.990	0.010	99.5			
bio17	0.994	0.006	176.4	0.979	0.021	47.6			
bio18	0.985	0.015	67.9	0.969	0.031	32.0			
bio19	0.983	0.017	58.2	0.978	0.022	45.5			
elevation	0.532	0.468	2.1	0.322	0.678	1.5	0.194	0.806	1.2

Table S4. Validation of the SDM's prediction results against the archaeological assemblages that were not confidentially assigned to a single time interval of the MIS14-MIS11 period. The predicted values indicate habitat suitability within a range of 0 (low) and 1 (high) throughout all the MIS substages considered. The coloured cells display whether the site's suitability reached the threshold of 0.118, computed as the 5%-percentile of the presence values. Green colours show suitable conditions and red non-suitable conditions. In 33 out of the 35 cases, suitable conditions are predicted in at least one of the time intervals corresponding to the stage to which the assemblage is correlated.

Locality	Unit	MIS Correlation	Suitability prediction during MIS										
			11ab	11c	11de	12a	12b	12c	13a	13b	13c	14ac	14d
Cagny-La-Garenne II	Levels I & H	MIS 11	0.79	0.84	0.80	0.74	0.73	0.75	0.82	0.80	0.79	0.76	0.79
Ariendorf	1	MIS 11	0.41	0.61	0.43	0.22	0.19	0.28	0.51	0.45	0.41	0.31	0.42
Schoeningen	Scho 12	MIS 11	0.22	0.79	0.26	0.03	0.02	0.07	0.46	0.29	0.22	0.10	0.23
Bilzingsleben II	II	MIS 11	0.15	0.60	0.17	0.02	0.02	0.05	0.31	0.20	0.14	0.07	0.15
Atapuerca Gran Dolina	TD10-3	MIS 11	0.73	0.90	0.76	0.35	0.29	0.49	0.84	0.77	0.73	0.58	0.73
Malagrotta	-	MIS 11	0.85	0.95	0.86	0.67	0.67	0.75	0.90	0.86	0.84	0.79	0.84
Terra Amata	C1a	MIS 11	0.48	0.85	0.52	0.19	0.16	0.28	0.67	0.55	0.47	0.32	0.48
Terra Amata	C1b	MIS 11	0.48	0.85	0.52	0.19	0.16	0.28	0.67	0.55	0.47	0.32	0.48
Ambrona	AS1-4	MIS 11	0.61	0.70	0.62	0.34	0.28	0.44	0.69	0.64	0.61	0.49	0.60
Beeches Pit	6	MIS 11	0.66	0.98	0.69	0.21	0.17	0.33	0.87	0.73	0.65	0.42	0.66
Beeches Pit	5	MIS 11	0.66	0.98	0.69	0.21	0.17	0.33	0.87	0.73	0.65	0.42	0.66
Beeches Pit	7	MIS 11	0.66	0.98	0.69	0.21	0.17	0.33	0.87	0.73	0.65	0.42	0.66
Le Grande Vallée	U5a - U5e	MIS 11	0.51	0.51	0.51	0.45	0.45	0.48	0.52	0.52	0.51	0.49	0.51
Menez-Dregan I	Layer 7	MIS 11	0.02	0.02	0.02	0.03	0.03	0.03	0.02	0.02	0.02	0.03	0.02
Aldène	TU II	MIS 11	0.74	0.78	0.75	0.57	0.54	0.64	0.78	0.76	0.74	0.68	0.74
Schladebach/Wallendorf	-	MIS 11	0.16	0.68	0.18	0.02	0.02	0.05	0.35	0.21	0.16	0.07	0.16
Uichteritz	Middle Gravel	MIS 11a-c	0.15	0.65	0.17	0.02	0.02	0.05	0.33	0.20	0.15	0.07	0.16
Kärlich	Level H	MIS 12	0.44	0.59	0.45	0.26	0.23	0.32	0.52	0.47	0.44	0.35	0.44
Boxgrove	6b, 8a, 8b,	MIS 12	0.19	0.43	0.20	0.05	0.04	0.08	0.29	0.22	0.18	0.11	0.19
Atapuerca Gran Dolina	TD10-4	MIS 12	0.73	0.90	0.76	0.35	0.29	0.49	0.84	0.77	0.73	0.58	0.73
Caune de l'Arago	CM_III	MIS 12	0.80	0.91	0.81	0.58	0.54	0.67	0.87	0.82	0.80	0.71	0.80
Cimitero di Atella	L	MIS 12	0.94	0.98	0.94	0.83	0.81	0.89	0.96	0.95	0.94	0.91	0.94
Aldène	TU I	MIS 12	0.74	0.78	0.75	0.57	0.54	0.64	0.78	0.76	0.74	0.68	0.74
Atapuerca Sima de los Huesos	LU 6	MIS 12	0.73	0.90	0.76	0.35	0.29	0.49	0.84	0.77	0.73	0.58	0.73

Locality	Unit	MIS Correlation	Suitability prediction during MIS										
			11ab	11c	11de	12a	12b	12c	13a	13b	13c	14ac	14d
High Lodge	Bed E	MIS 12c	0.68	0.99	0.71	0.23	0.18	0.35	0.88	0.76	0.67	0.44	0.68
Cueva de Bolomor	Fase I	MIS 13	0.33	0.57	0.33	0.17	0.15	0.22	0.41	0.34	0.31	0.24	0.31
Boxgrove	4b	MIS 13	0.19	0.43	0.20	0.05	0.04	0.08	0.29	0.22	0.18	0.11	0.19
Boxgrove	5a	MIS 13	0.19	0.43	0.20	0.05	0.04	0.08	0.29	0.22	0.18	0.11	0.19
Boxgrove	4c	MIS 13	0.19	0.43	0.20	0.05	0.04	0.08	0.29	0.22	0.18	0.11	0.19
Caune de l‘Arago	CM_II	MIS 13	0.80	0.91	0.81	0.58	0.54	0.67	0.87	0.82	0.80	0.71	0.80
Valdoe	Slindon sands	MIS 13	0.12	0.27	0.13	0.04	0.03	0.06	0.19	0.14	0.12	0.08	0.12
Happisburgh 1	Organic mud and grey sands	MIS 13	0.38	0.94	0.42	0.05	0.04	0.11	0.68	0.48	0.36	0.16	0.38
High Lodge	Bed C	MIS 13	0.68	0.99	0.71	0.23	0.18	0.35	0.88	0.76	0.67	0.44	0.68
Waverly Wood	-	MIS 13	0.67	0.90	0.70	0.38	0.34	0.48	0.80	0.72	0.67	0.53	0.68
Caune de l‘Arago	CM_I	MIS 14	0.80	0.91	0.81	0.58	0.54	0.67	0.87	0.82	0.80	0.71	0.80

Table S5. Hunter-gatherers in Binford's dataset[64] living above parallel 30 N. Net Primary Productivity (NPP) estimates based on the Miami model[65]. This table is an Excel spreadsheet contained in an individual tab in the Excel file available for download in "Supplementary Information."

Table S6. Results of the k-means cluster analysis used to classify the hunter-gatherer groups according to their procurement strategy. Setting the number of clusters to two divides the sample into a 'hunter-gatherer' group and a 'fishers' group. However, the 'hunter-gatherer' group is rather heterogeneous concerning the relative weight of hunting in its diet. A more robust classification is obtained when the number of clusters is set to three and the sample is divided among 'hunters', 'fishers' and 'gatherers'.

Variable	Mean Cluster 1	Mean Cluster 2	Mean Cluster 3	SS Inter cluster	d.f	SS intra cluster	d.f.	F
Two Clusters								
%Gathering	11.83	32.29	-	21410.5	1	72847.2	213	62.6
%Hunting	20.81	47.9	-	37691.0	1	61439.6	213	130.7
%Fishing	67.35	19.76	-	115916.4	1	41098.2	213	600.8
Three clusters								
%Gathering	11.01	50.37	11.76	72042.0	2	22216.8	212	343.7
%Hunting	65.29	32.80	20.68	72150.7	2	26979.8	212	283.5
%Fishing	23.68	16.83	67.54	116760.0	2	40254.5	212	307.5

Figure S1. Relationship between hunter-gatherer population density (D) and net primary productivity (NPP). Green squares represent groups classified as ‘gatherers’, and red circles represent ‘hunters’. Solid squares and dots correspond to groups with domesticated horses.

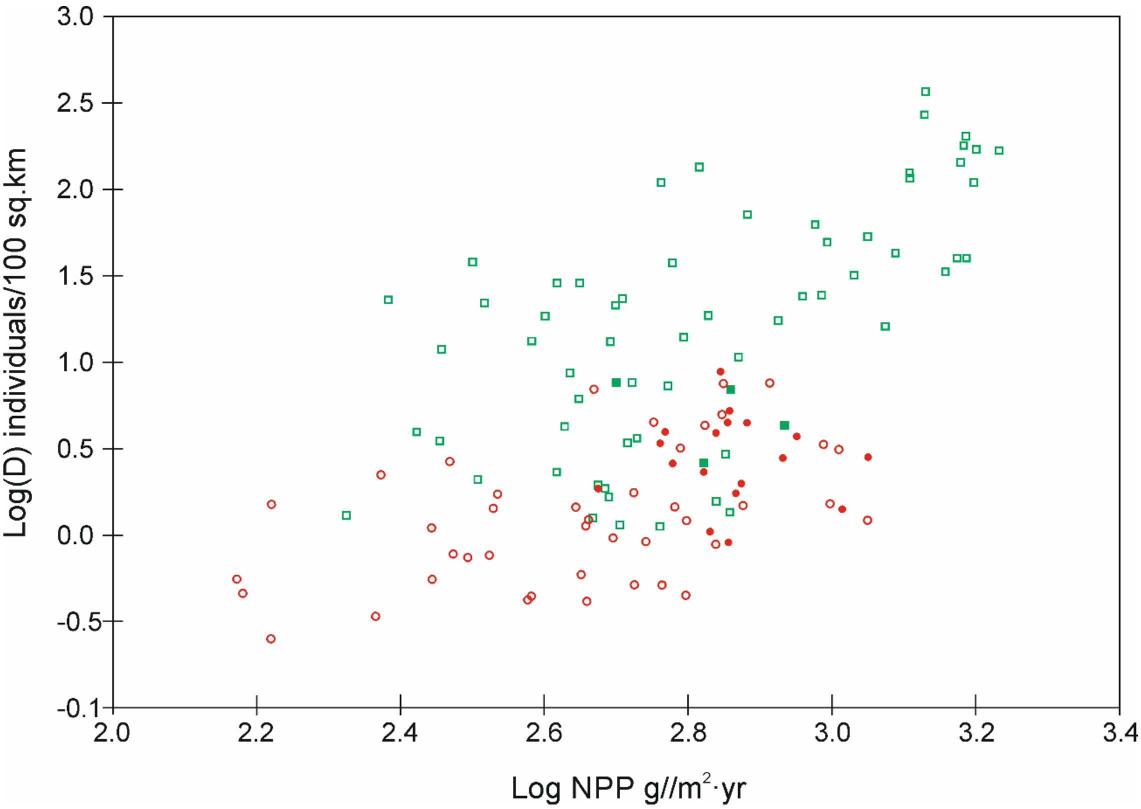

Figure S2. Variation of the area suitable for humans along the MIS14 to MIS11 period. The map represents the number of time periods each pixel was classified as "suitable" by the niche model (5%-percentile of predicted values at observed sites). Thereafter dark-green areas are considered continuously habitable core areas, while light-green areas are only occasionally within the habitable range during temporary favourable conditions. The map was created in QGIS 3.22 (https://www.qgis.org).

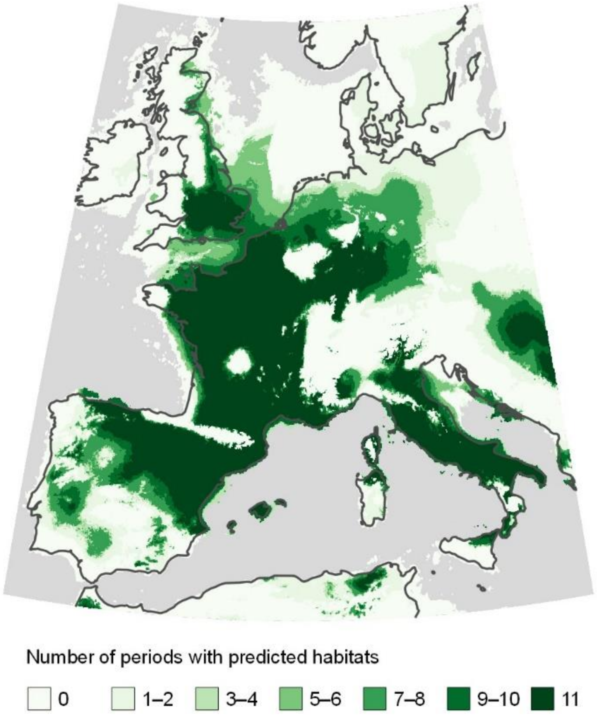

Figure S3. Pairwise correlation matrix (Pearson's r) between environmental variables.

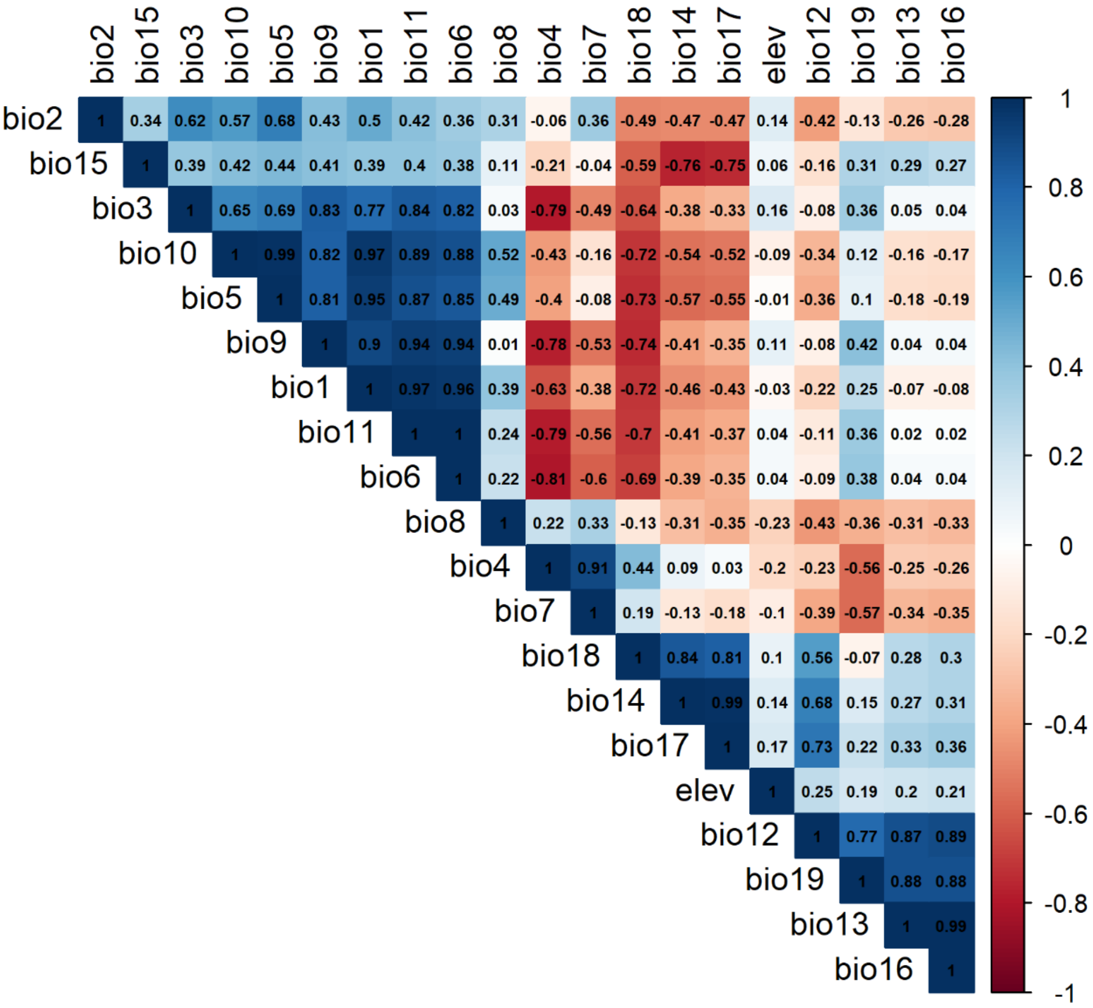

Figure S4. Effect of different sampling sizes and strategies on the background sample. In (a), we performed an analysis to test the effect of different sample sizes between n=1 and n=100,000. Thereafter, the distribution of the sampled values can be considered stable when the sample exceeds the size of n>1,000. In our study, we decided to use n=10,000. In (b), we tested the impact of two alternative sampling strategies based on a sample size of n=10,000. The random strategy draws the sample randomly from all 11 time intervals. For the stratified strategy, the number of samples drawn from each time interval is weighted by the number of archaeological sites associated with the respective interval. MIS 11ab, for example, features 12 of the 33 sites considered in this study, so we sampled 12/33*10,000=3,636 values from this interval. We conclude that the strategies exert a minor impact on the distribution of the background samples. In our study, we decided to apply the random sampling strategy.

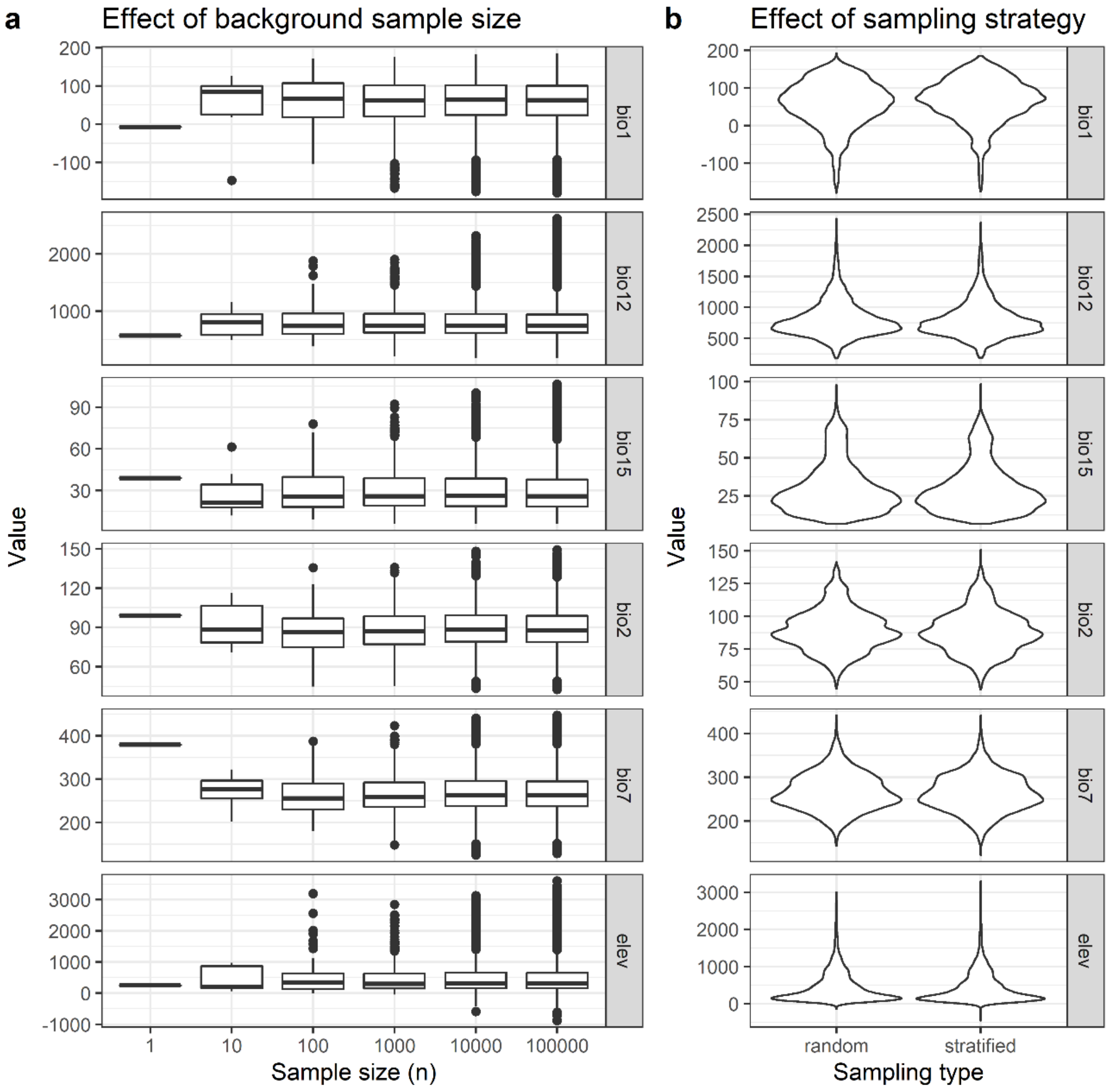

References cited in Supplementary Tables S1 and S2

1 Auguste, P. Ëvolution des peuplements mammaliens en Europe de Nord-Ouest durant le Pleistocene moyen et Supérieur. Le cas de la France Septentrionale. *Quaternaire* **20**, 527-550 (2009).

2 Koenigswald, W. V. & Heinrich, W.-D. Mittelpleistozäne Säugetierfaunen Mitteleuropa - der Versuch einer bioestratigraphischen Zuordnung. *Kaupia* **9**, 53-112 (1999).

3 Turner, E. Middle and Late Pleistocene Macrofaunas of the Neuwied Basin Region (Rhineland-Palatinate) of West Germany. *Jarbuch des Römisch-Germaninchen Zentralmuseums Mainz* **37**, 133-403 (1990).

4 Haidle, M. N. & Pawlik, A. F. The earliest settlement of Germany: Is there anything out there? *Quaternary International* **223-224**, 143-153, doi:DOI: 10.1016/j.quaint.2010.02.009 (2010).

5 Conard, N. J. *et al.* Excavations at Schöningen and paradigm shifts in human evolution. *Journ. Hum. Evol.* **89**, 1-17, doi:http://dx.doi.org/10.1016/j.jhevol.2015.10.003 (2015).

6 Kolfschoten, T. v. The Palaeolithic locality Schöningen (Germany): A review of the mammalian record. *Quaternary International* **326-327**, 469-480, doi:http://dx.doi.org/10.1016/j.quaint.2013.11.006 (2014).

7 Richter, D. & Krbetschek, M. The age of the Lower Paleolithic occupation at Schöningen. *Journ. Hum. Evol.* **89**, 46-56, doi:http://dx.doi.org/10.1016/j.jhevol.2015.06.003 (2015).

8 Mania, D. in *The first Europeans* (eds E. Carbonell, J. M. Bermúdez de Castro, J. L. Arsuaga, & X. P. Rodríguez) 137-150 (Aldecoa, 1998).

9 Rodríguez, J. *et al.* One million years of cultural evolution in a stable environment at Atapuerca (Burgos, Spain). *Quaternary Science Reviews* **30**, 1396-1412, doi:10.1016/j.quascirev.2010.02.021 (2011).

10 Ceruleo, P. *et al.* New chronological framework (MIS 13–9) and depositional context for the lower Palaeolithic sites north-west of Rome: Revisiting the early hominin in central Italy. *Quaternary International* **510**, 119-132, doi:https://doi.org/10.1016/j.quaint.2019.01.001 (2019).

11 Valensi. Évolution des peuplements de grands mammifères en Europe Méditerranéene Occidentale durant le Pleistocene moyen et supérieur. Un exemple régional: Les Alpes du sud Françaises et italiennes. *Quaternarire* **20**, 551-567 (2009).

12 Palombo, M. R. & Valli, A. M. F. Remarks on the biochronology of mammalian faunal complexes from the Pliocene to the middle Pleistocene in France. *Geologica Romana* **37**, 145-163 (2003-2004).

13 Falguères, C. *et al.* The Lower Acheulian site of Ambrona, Soria (Spain): ages derived from a combined ESR/U-series model. *Journ. Archaeol. Sci.* **33**, 149-157, doi:http://dx.doi.org/10.1016/j.jas.2005.07.006 (2006).

14 Santonja, M. *et al.* Ambrona revisited: The Acheulean lithic industry in the Lower Stratigraphic Complex. *Quaternary International* **480**, 95-117, doi:https://doi.org/10.1016/j.quaint.2017.01.021 (2018).

15 Preece, R. C., Gowlett, J. A. J., Parfitt, S. A., Bridgland, D. R. & Lewis, S. G. Humans in the Hoxnian: habitat, context and fire use at Beeches Pit, West Stow, Suffolk, UK. *Journal of Quaternary Science* **21**, 485-496, doi:10.1002/jqs.1043 (2006).

16 Hérisson, D. *et al.* Between the northern and southern regions of Western Europe: The Acheulean site of La Grande Vallée (Colombiers, Vienne, France). *Quaternary International* **411**, 108-131, doi:https://doi.org/10.1016/j.quaint.2015.12.100 (2016).

17 Mercier, N. *et al.* Nouvelles données chronologiques pour le site de Menez-Dregan 1 (Bretagne): l'apport de la thermoluminescence New chronological data of the site of Menez-Dregan 1 (Brittany) : contribution from the thermoluminescence dating method. . *Quaternaire*, 253-261 (2004).

18 Monnier, J.-L. *et al.* Menez-Dregan 1 (Plouhinec, Finistère, France) : un site d’habitat du Paléolithique inférieur en grotte marine. Stratigraphie, structures de combustion,

industries riches en galets aménagés. *L'Anthropologie* **120**, 237-262, doi:https://doi.org/10.1016/j.anthro.2016.05.003 (2016).
19 Ravon, A.-L., Monnier, J.-L. & Laforge, M. Menez-Dregan I, layer 4: A transitional layer between the Lower and Middle Palaeolithic in Brittany. *Quaternary International* **409**, 92-103, doi:https://doi.org/10.1016/j.quaint.2015.07.066 (2016).
20 Rossoni-Notter, E., Notter, O., Simone, S. & Simon, P. Acheulean technical behaviors in Aldène cave (Cesseras, Hérault, France). *Quaternary International* **409**, 149-173, doi:https://doi.org/10.1016/j.quaint.2015.11.016 (2016).
21 Lauer, T. & Weiss, M. Timing of the Saalian- A nd Elsterian glacial cycles and the implications for Middle-Pleistocene hominin presence in central Europe. *Scientific Reports* **8**, doi:10.1038/s41598-018-23541-w (2018).
22 Sesé, C. & Soto, E. in *Patrimonio Paleontológico de la Comunidad de Madrid* (ed J. Morales) 216-243 (Consejería de Educación. Comunidad de Madrid, 2002).
23 Yravedra, J. *et al.* Cut marks on the Middle Pleistocene elephant carcass of Áridos 2 (Madrid, Spain). *Journ. Archaeol. Sci.* **37**, 2469-2476, doi:https://doi.org/10.1016/j.jas.2010.05.007 (2010).
24 Ashton, N. The human occupation of Britain during the Hoxnian Interglacial. *Quaternary International* **409**, 41-53, doi:https://doi.org/10.1016/j.quaint.2015.11.055 (2016).
25 Pereira, A. *et al.* Integrated geochronology of Acheulian sites from the southern Latium (central Italy): Insights on human-environment interaction and the technological innovations during the MIS 11-MIS 10 period. *Quaternary Science Reviews* **187**, 112-129, doi:https://doi.org/10.1016/j.quascirev.2018.03.021 (2018).
26 Ollé, A. *et al.* The Middle Pleistocene site of La Cansaladeta (Tarragona, Spain): Stratigraphic and archaeological succession. *Quaternary International* **393**, 137-157, doi:https://doi.org/10.1016/j.quaint.2015.08.053 (2016).
27 Ashton, N., Lewis, S. G., Parfitt, S. A., Penkman, K. E. H. & Russell Coope, G. New evidence for complex climate change in MIS 11 from Hoxne, Suffolk, UK. *Quaternary Science Reviews* **27**, 652-668, doi:https://doi.org/10.1016/j.quascirev.2008.01.003 (2008).
28 Palombo, M. R., Azanza, B. & Alberdi, M. T. Italian Mammal biochronology from the Latest Miocene to the Middle Pleistocene: A multivariate approach. *Geologica Romana* **36**, 335-368 (2000-2002).
29 Sala, B. & Masini, F. Late Pliocene and Pleistocene small mammal chronology in the Italian peninsula. *Quaternary International* **160**, 4-16 (2007).
30 Pereira, A. *et al.* 40Ar/39Ar and ESR/U-series dates for Guado San Nicola, Middle Pleistocene key site at the Lower/Middle Palaeolithic transition in Italy. *Quaternary Geochronology* **36**, 67-75, doi:https://doi.org/10.1016/j.quageo.2016.08.005 (2016).
31 Antoine, P. & Limondin-Lozouet, N. Identification of MIS 11 Interglacial tufa deposit in the Somme valley (France): new results from the Saint-Acheul fluvial sequence. *Quaternaire* **15**, 41-52 (2004).
32 Lauer, T. *et al.* The Middle Pleistocene fluvial sequence at Uichteritz, central Germany: Chronological framework, paleoenvironmental history and early human presence during MIS 11. *Geomorphology* **354**, 107016, doi:https://doi.org/10.1016/j.geomorph.2019.107016 (2020).
33 Ashton, N. M. *et al.* Excavations at the Lower Palaeolithic site at East Farm, Barnham, Suffolk 1989-92. *Journal-of-the-Geological-Society-(London).* **151**, 599-605. (1994).
34 Limondin-Lozouet, N. *et al.* Oldest evidence of Acheulean occupation in the Upper Seine valley (France) from an MIS 11 tufa at La Celle. *Quaternary International* **223**, 299-311, doi:10.1016/j.quaint.2009.10.013 (2010).
35 Daura, J. *et al.* New Middle Pleistocene hominin cranium from Gruta da Aroeira (Portugal). *Proceedings of the National Academy of Sciences*, doi:10.1073/pnas.1619040114 (2017).

36 Daura, J. *et al.* A 400,000-year-old Acheulean assemblage associated with the Aroeira-3 human cranium (Gruta da Aroeira, Almonda karst system, Portugal). *Comptes Rendus Palevol* **17**, 594-615, doi:https://doi.org/10.1016/j.crpv.2018.03.003 (2018).
37 Connet, N., Soriano, S., Bertran, P., Lhomme, V. & Debenham, N. A 400,000 years old milestone of the Acheulian technocomplex in Central-Western France at Londigny (Charente). *Journal of Archaeological Science: Reports* **30**, 102225, doi:https://doi.org/10.1016/j.jasrep.2020.102225 (2020).
38 Kolfschoten, T. V. & Turner, E. in *The early Middle Pleistocene in Europe* (ed C. Turner) 227-253 (Taylor & Francis, 1996).
39 García -Medrano, P., Ollé, A., Ashton, N. M. & Roberts, M. B. The Mental Template in Handaxe Manufacture: New Insights into Acheulean Lithic Technological Behavior at Boxgrove. *Journal of Archaeological Method and Theory*, doi:https://doi.org/10.1007/s10816-018-9376-0 (2018).
40 Falguères, C. *et al.* New ESR and U-series dating at Caune de l'Arago, France: A key-site for European Middle Pleistocene. *Quaternary Geochronology* **30, Part B**, 547-553, doi:http://dx.doi.org/10.1016/j.quageo.2015.02.006 (2015).
41 Hanquet, C. & Desclaux, E. Analyse paléoécologique des communautés de micromammifères de la Caune de l'Arago (Tautavel, France) dans le contexte des migrations de faunes en Europe méridionale au cours du Pléistocène moyen *Quaternaire* **22**, 35-45 (2011).
42 Magniez, P., Moigne, A.-M., Testu, A. & de Lumley, H. Biochronologie des Mammifères quaternaires. Apport des Cervidae du site Pléistocène moyen de la Caune de l'Arago (Tautavel, Pyrénées-orientales, France) », Quaternaire, vol. 24/4 | 2013, 477-502. *Quaternaire* **24**, 477-502 (2013).
43 Abruzzese, C., Aureli, D. & Rocca, R. Assessment of the Acheulean in Southern Italy: New study on the Atella site (Basilicata, Italy). *Quaternary International* **393**, 158-168, doi:https://doi.org/10.1016/j.quaint.2015.06.005 (2016).
44 Rocca, R., Aureli, D. & Abruzzese, C. « Cimitero di Atella », Chronique des activités archéologiques de l'École française de Rome Online, Italie du Sud. doi: https://doi.org/10.4000/cefr.1910 (2018).
45 Aranburu, A., Arsuaga, J. L. & Sala, N. The stratigraphy of the Sima de los Huesos (Atapuerca, Spain) and implications for the origin of the fossil hominin accumulation. *Quaternary International*, doi:http://dx.doi.org/10.1016/j.quaint.2015.02.044 (2015).
46 Carbonell, E. *et al.* Les premiers comportements funéraires auraient-ils pris place à Atapuerca, il y a 350 000 ans ? *L'Anthropologie* **107**, 1-14, doi:https://doi.org/10.1016/S0003-5521(03)00002-5 (2003).
47 Demuro, M., Arnold, L. J., Aranburu, A., Sala, N. & Arsuaga, J. L. New bracketing luminescence ages constrain the Sima de los Huesos hominin fossils (Atapuerca, Spain) to MIS 12. *Journ. Hum. Evol.* **131**, 76-95, doi:10.1016/j.jhevol.2018.12.003 (2019).
48 Tuffreau, A. Les fouilles paléolithiques de Cagny-Cimetière (Somme). *Revue archéologique de Picardie*, 5-16 (1980).
49 Nicoud, E. *et al.* Preliminary data from Valle Giumentina Pleistocene site (Abruzzo, Central Italy): A new approach to a Clactonian and Acheulian sequence. *Quaternary International* **409**, 182-194, doi:https://doi.org/10.1016/j.quaint.2015.08.080 (2016).
50 Villa, V. *et al.* A MIS 15-MIS 12 record of environmental changes and Lower Palaeolithic occupation from Valle Giumentina, central Italy. *Quaternary Science Reviews* **151**, 160-184, doi:https://doi.org/10.1016/j.quascirev.2016.09.006 (2016).
51 Lewis, S. G. *et al.* Human occupation of northern Europe in MIS 13: Happisburgh Site 1 (Norfolk, UK) and its European context. *Quaternary Science Reviews* **211**, 34-58, doi:https://doi.org/10.1016/j.quascirev.2019.02.028 (2019).
52 Lewis, S. G., Ashton, N., Hoare, P. G. & Parfitt, S. Human occupation of Northern Europe in MIS 13: a response to comments by Gibbard et al. (2019). *Quaternary Science Reviews* **223**, 105851, doi:https://doi.org/10.1016/j.quascirev.2019.07.033 (2019).

53 Fernández, J., Guillem, P. & Martínez, R. in *Actas del 3er congreso de Arqueología Peninsular.* (ed V. Olivera) 82-100.
54 Rivals, F. & Blasco, R. Presence of Hemitragus aff. cedrensis (Mammalia, Bovidae) in the Iberian Peninsula: Biochronological and biogeographical implications of its discovery at Bolomor Cave (Valencia, Spain). *Comptes Rendus Palevol* **7**, 391-399, doi:DOI: 10.1016/j.crpv.2008.05.003 (2008).
55 Roberts, M. B. & Parfitt, S. A. Boxgrove. A middle Pleistocene hominid site at Eartham Quarry, Boxgrove, West Sussex. *English Heritage. Archaeological Report* **17** (1999).
56 Roberts, M., Pope, M., Maxted, A. & Jones, P. The Valdoe: Archaeology of a Locality within the Boxgrove Palaeolandscape, West Sussex. *Proceedings of the Prehistoric Society (London)* **75**, 239-263, doi:10.1017/S0079497X00000360 (2009).
57 Voinchet, P. *et al.* New chronological data (ESR and ESR/U-series) for the earliest Acheulian sites of north-western Europe. *Journal of Quaternary Science* **30**, 610-622, doi:10.1002/jqs.2814 (2015).
58 Gibbard, P. L., Hughes, P. D. & West, R. G. Human occupation of northern Europe in MIS 13: Happisburgh Site 1 (Norfolk, UK) and its European context: A response to Lewis et al. (2019). *Quaternary Science Reviews* **223**, doi:10.1016/j.quascirev.2019.07.026 (2019).
59 Keen, D. H., Hardaker, T. & Lang, A. T. O. A Lower Palaeolithic industry from the Cromerian (MIS 13) Baginton Formation of Waverley Wood and Wood Farm Pits, Bubbenhall, Warwickshire, UK. *Journal of Quaternary Science* **21**, 457-470, doi:10.1002/jqs.1047 (2006).
60 Aureli, D. *et al.* Mode 1 or mode 2? "Small tools" in the technical variability of the European Lower Palaeolithic: The site of Ficoncella (Tarquinia, Lazio, central Italy). *Quaternary International* **393**, 169-184, doi:https://doi.org/10.1016/j.quaint.2015.07.055 (2016).
61 Antoine, P. *et al.* Dating the earliest human occupation of Western Europe: New evidence from the fluvial terrace system of the Somme basin (Northern France). *Quaternary International* **370**, 77-99, doi:https://doi.org/10.1016/j.quaint.2014.08.012 (2015).
62 Railsback, L. B., Gibbard, P. L., Head, M. J., Voarintsoa, N. R. G. & Toucanne, S. An optimized scheme of lettered marine isotope substages for the last 1.0 million years, and the climatostratigraphic nature of isotope stages and substages. *Quaternary Science Reviews* **111**, 94-106, doi:https://doi.org/10.1016/j.quascirev.2015.01.012 (2015).
63 Gamisch, A. Oscillayers: A dataset for the study of climatic oscillations over Plio-Pleistocene time-scales at high spatial-temporal resolution. *Global Ecology and Biogeography* **28**, 1552-1156, doi:10.1111/geb.12979 (2019).
64 Binford, L. R. *Constructing Frames of Reference: An Analytical Method for Archaeological Theory Building Using Ethnographic and Environmental Data Set.* (University of California Press, 2001).
65 Tallavaara, M., Luoto, M., Korhonen, N., Järvinen, H. & Seppä, H. Human population dynamics in Europe over the Last Glacial Maximum. *Proceedings of the National Academy of Sciences* **112**, 8232-8237, doi:https://doi.org/10.1073/pnas.1503784112 (2015).
